# First Direct Evidence for a Structurally Stable Adhesion Between the Perialgal Vacuole Membrane and Host Mitochondria in the *Paramecium*-*Chlorella* Endosymbiosis

**DOI:** 10.3390/biom16040561

**Published:** 2026-04-10

**Authors:** Masahiro Fujishima, Sho Nishiyama

**Affiliations:** 1 Research Center for Thermotolerant Microbial Resources, Yamaguchi University, Yoshida 1677-1, Yamaguchi 753-8515, Japan; 2 Institute of Environmental Radioactivity, Fukushima University, Kanayagawa 1, Fukushima 960-1296, Japan; 3 Department of Biology and Chemistry, Faculty of Science, Yamaguchi University, Yoshida 1677-1, Yamaguchi 753-8512, Japan

**Keywords:** endosymbiotic *Chlorella*, PV membrane, mitochondrial membrane, mitochondria–PV membrane complex, *Paramecium bursaria*, BODIPY FL C_5_-ceramide, mitochondria-specific monoclonal antibody

## Abstract

Physical integration between endosymbiotic algae and host mitochondria is a recurring feature across photosynthetic symbioses, yet the structural nature of this association has remained unresolved. In the ciliate *Paramecium bursaria*, each endosymbiotic *Chlorella* cell is enclosed by a perialgal vacuole (PV) membrane consistently surrounded by host mitochondria, suggesting a conserved architecture for metabolic interaction. Although transmission electron microscopy has shown close membrane apposition, it has remained unclear whether this reflects incidental proximity or a reinforced adhesion. Here, we provide direct evidence that the PV membrane and host mitochondrial membrane form a stable physical association. Using discontinuous Percoll centrifugation, we isolated intact units in which *Chlorella* and mitochondria co-sedimented, indicating that their association withstands mechanical disruption. By fluorescently labeling the PV and mitochondrial membranes with BODIPY FL C_5_-ceramide (BC_5_C), together with a mitochondria-specific monoclonal antibody and DAPI, we visualized the PV membrane under light microscopy and demonstrated that the mitochondrial–PV membrane complex persists after homogenization and centrifugation. As expected from the membrane-insertion behavior of BC_5_C, this fluorescent labeling revealed that the PV–mitochondrial membrane association is structurally reinforced rather than incidental, providing a mechanistic framework for understanding how *Chlorella* cells are stably positioned beneath the host cortex.

## 1. Introduction

Photosynthetic endosymbiosis represents one of the most consequential innovations in eukaryotic evolution, enabling heterotrophic hosts to acquire photosynthetic capacity by incorporating phototrophic partners. The ciliate *Paramecium bursaria* is a well-established model for studying this process, as it harbors several hundred endosymbiotic *Chlorella* cells [1], each enclosed within a perialgal vacuole (PV) that physically separates the alga from the host cytoplasm. Although *P. bursaria* can survive without algae, the acquisition of photosynthetic symbionts confers substantial ecological advantages, including enhanced starvation tolerance [2,3], increased thermal [4] and hypoxic resistance [5], and access to photosynthetically derived maltose and oxygen [6,7,8]. Thus, the ability to maintain algae in a stable cortical position is a key adaptation that enhances host fitness.

After escaping from the host digestive vacuole (DV) by the budding of the DV membrane, compatible algae migrate to the subcortical region and become immobilized. During this transition, the DV-derived membrane differentiates into the PV membrane, loses acid phosphatase activity, and prevents lysosomal fusion, thereby ensuring algal survival [9]. Immobilization beneath the cortex is essential not only for protection from digestion but also for reliable partitioning of algae into daughter cells during host division [10] and for shielding the symbionts from ultraviolet radiation [11]. In contrast, infection-incapable *Chlorella* species fail to establish this cortical attachment and are ultimately digested [12].

The consistent positioning of endosymbiotic algae near the host mitochondria just beneath the host cell surface is not unique to *P. bursaria*. Similar spatial arrangements have been documented in diverse protists [13] and other photosynthetic endosymbioses, such as coral–Symbiodiniaceae associations [14,15], sea anemones [16], giant clams (*Tridacna*) [17] and even kleptoplastic gastropods [18]. These observations suggest that the close apposition of host mitochondria to intracellular phototrophs may represent a recurrent architectural motif that facilitates metabolic exchange in photosynthetic symbioses.

In *P. bursaria*, transmission electron microscopy (TEM) studies have repeatedly shown that the PV membrane appears to be in direct contact with the outer membrane of host mitochondria [19,20]. Cryofixation analyses further revealed that mitochondria not only contact the PV membrane but also extend toward the algal cell wall, forming networks interconnected with other mitochondria and the host endoplasmic reticulum [21]. However, TEM alone cannot determine whether this association reflects incidental proximity or a structurally stable adhesion. This distinction is critical because a naturally occurring mutant isolated from the field exhibits defective cortical attachment of *Chlorella*, resulting in unstable inheritance of symbionts during host cell division and eventual loss of algae [10]. This phenotype provides strong evidence that PV–mitochondrion adhesion is essential for stable symbiont retention.

Physiological observations also indicate that this association is dynamically maintained. When living *P. bursaria* cells are subjected to centrifugal force, PV-enclosed algae detach from the cortex and accumulate at the posterior end of the cell, yet they reattach to their original subcortical positions within 15 min after centrifugation ceases [22]. This rapid recovery implies the presence of an active mechanism that restores PV–cortex association, but the structural basis of this process has remained unresolved.

A definitive test of whether the PV membrane and host mitochondria are truly adhered is to determine whether their association persists after mechanical disruption of host cells. If the two membranes are tightly attached rather than merely adjacent, they should remain connected even after homogenization and centrifugation. In this study, we examined whether mitochondria remain associated with isolated PV-enclosed *Chlorella* cells following mechanical disruption by combining a newly developed isolation condition for symbiotic *Chlorella* cells from *P. bursaria* homogenates and a newly developed fluorescence labeling technique for the PV membrane and the host mitochondrial membrane. Using this combined approach, we provide direct evidence that the PV–mitochondrion association represents a physically stable membrane interaction rather than incidental proximity. Similar host-derived membrane interfaces have been described in diverse intracellular symbioses, where they function as dynamic platforms for metabolic exchange and cellular integration [23,24] and may represent a broader class of membrane contact structures in eukaryotic cells [25,26].

## 2. Materials and Methods

### 2.1. Strains and Cultures

The *Chlorella variabilis*-bearing (symbiotic) *Paramecium bursaria* strain Yad1g1N (syngen 1, mating type I) and the *Chlorella*-free (aposymbiotic) strain Yad1w were used in this study. The original Yad1g strain was collected from a pond at Yamaguchi University, Yoshida Campus, Japan, by Ayako Nishimura in 2004, and the aposymbiotic strain Yad1w was generated from Yad1g [9]. The symbiotic strain Yad1g1N was later established by infecting Yad1w cells with cloned symbiotic *C. variabilis* strain 1N [20]. The *C. variabilis* strain 1N was cloned by Dr. Miho Nakahara-Tsubota from *P. bursaria* strain OS1g (syngen 1, mating type I), originally collected by Dr. Isoji Miwa (Ibaraki University) from Itako City, Japan, in 2002.

Symbiotic and aposymbiotic paramecia were cultivated in glass test tubes (18 × 180 mm) containing modified Dryl’s solution (MDS; KH_2_PO_4_ substituted for NaH_2_PO_4_·2H_2_O) [27,28] supplemented with 1.25% (*w*/*v*) fresh lettuce juice and 0.0001% (*w*/*v*) stigmasterol (Tama Biochemical Co., Ltd., Tokyo, Japan) at 25 ± 1 °C. The medium was inoculated with the non-pathogenic *Klebsiella pneumoniae* strain 6081 one day before use [28]. For routine culture, several hundred cells were inoculated into 2 mL of medium, and 2 mL of fresh medium was added daily for 12 days. One day after the final feeding, cultures reached early stationary phase, and cells at this stage were used for all experiments.

All strains were maintained in the Fujishima laboratory (Yamaguchi University, Japan) and subsequently deposited in the National BioResource Project *Paramecium* (NBRP–*Paramecium*, http://nbrpcms.nig.ac.jp/paramecium/, accessed on 31 August 2014).

### 2.2. Isolation of Symbiotic Algae Possessing PV Membranes and Mitochondria by Discontinuous Percoll Density Gradient Centrifugation

Cultures of *P. bursaria* (approximately 600 mL) in early stationary phase were filtered through two layers of Kimwipes (Kimberly-Clark, Kokusan Co., Ltd., Saitama City, Japan) to remove debris and centrifuged at 300× *g* for 3 min at room temperature using an oil-test centrifuge (H-210A, Kokusan Co., Ltd., Saitama City, Japan) with 100 mL oil-separation glass tubes. The pellet was washed once with ice-chilled MDS under the same conditions, resuspended in 2.5 mL of ice-cold homogenization buffer (200 mM sucrose, 10 mM Na/K phosphate buffer, pH 6.5), and transferred to a pre-chilled 1 mL Teflon homogenizer. Cells were gently disrupted by five strokes of the pestle on ice.

The homogenate was layered onto a discontinuous Percoll (2.5 mL of 75% (*v*/*v*) Percoll overlaid with 2.5 mL of 45% (*v*/*v*) Percoll) prepared in 12 mL centrifuge tubes (Nalgene 3110-0120PK, Thermo Fisher Scientific, Rochester, NY, USA) using stock isotonic Percoll (SIP; 100% (*v*/*v*) Percoll mixed with 2.5 M sucrose at 9:1). SIP was diluted with 250 mM sucrose to prepare the gradient. The Percoll used in this study was obtained from GE Healthcare (Uppsala, Sweden; lot no. 10067066). Centrifugation was performed at 600× *g* for 25 min at 4 °C using a TS-7 swing rotor in an RS-18IV centrifuge (TOMY SEIKO CO., Ltd., Tokyo, Japan). The green *Chlorella*-containing band at the 75%/45% interface was collected with a Pasteur pipette, counted using a hemocytometer, and stored at 4 °C until use.

### 2.3. Routine Isolation of Symbiotic Chlorella

For routine isolation of symbiotic algae for infection experiments, cultures of *P. bursaria* (approximately 600 mL) were filtered through two layers of Kimwipes and concentrated using a 50 mL plastic centrifuge tube fitted with a 15 µm nylon mesh. Cells were washed with MDS on the mesh, concentrated to 1 mL, transferred to a 1 mL Teflon homogenizer, and disrupted by 10 strokes of the pestle on ice. The homogenate was passed through a new mesh to remove debris while allowing *Chlorella* cells to pass into the filtrate. The filtrate was centrifuged at 4355× *g* for 30 s at 25 ± 1 °C (CR-12, TAITEC Corporation, Koshigaya City, Saitama Pref., Japan), washed three times with 1.5 mL of MDS, and stored at 4 °C in the dark until use.

### 2.4. Production of Monoclonal Antibodies Against P. bursaria Mitochondria

A mitochondrion-specific monoclonal antibody was produced by immunizing BALB/c mice (4–5 weeks old) with the symbiotic algae–mitochondria fraction obtained by discontinuous Percoll centrifugation. Mice received intraperitoneal injections of antigen three times at two-week intervals. For the first immunization, the antigen was mixed with an equal volume of BACTO Freund’s complete adjuvant (BD Difco^TM^, Franklin Lakes, NJ, USA). For the second and third immunizations, the antigen was mixed with BACTO Freund’s incomplete adjuvant (Difco). Hybridomas producing the desired antibody were screened by indirect immunofluorescence and limiting dilution [29]. The monoclonal antibody mAb-3G11E3F7 was used in this study. This antibody recognizes a mitochondrial antigen. However, the precise sub-mitochondrial localization (e.g., outer membrane, inner membrane, or matrix-facing) has not been determined, and the antigen itself remains unidentified.

Hybridoma production followed the institutional guidelines for animal use in research at Yamaguchi University.

### 2.5. Indirect Immunofluorescence Microscopy

*P. bursaria* cells in early stationary phase were air-dried on coverslips (4.5 × 24 mm, Matsunami Glass Ind., Ltd., Kishiwada City, Oosaka, Japan), fixed with cold 4% (*w*/*v*) paraformaldehyde in PBS (137 mM NaCl, 2.68 mM KCl, 8.1 mM Na_2_HPO_4_·12H_2_O, 1.47 mM KH_2_PO_4_, pH 7.2) for 15 min, treated with cold PBST (PBS containing 0.05% (*v*/*v*) Tween-20) for 10 min, and washed twice with PBS. Cells were incubated with hybridoma culture supernatant containing the primary antibody (mAb-3G11E3F7) for 60 min at room temperature, washed twice with PBS, and incubated with Alexa Fluor 488–conjugated goat anti-mouse IgG (1:1000; Molecular Probe, Inc., Eugene, OR, USA) for 60 min. After two PBS washes, cells were stained with 0.001% (*w*/*v*) DAPI (Dojindo Laboratories, Kumamoto, Japan) for 5 min and washed again. Samples were examined using DIC and fluorescence microscopy (BX60, Olympus Corporation, Hachiouji City, Japan). For isolated *Chlorella* enclosed by PV membranes and mitochondria, PBST washing after fixation was replaced with PBS washing.

### 2.6. Fluorescent Staining of the PV Membrane and Mitochondrial Membrane

The PV membrane surrounding symbiotic *Chlorella* and the mitochondrial membrane co-sedimented with the algae were stained with BODIPY FL C_5_-ceramide complexed to BSA (BC_5_C/BSA; B22650, Invitrogen, Carlsbad, CA, USA). A 50 μM stock solution was prepared in deionized water, stored at 4 °C, and diluted to 5 μM immediately before use. The diluted BC_5_C/BSA was added to the isolated *Chlorella*–mitochondria fractions and incubated for 30 min at room temperature in the dark before fluorescence microscopy. When required, DAPI was added to a final concentration of 0.001% (*w*/*v*). BC_5_C/BSA is known to be converted to BODIPY FL C_5_-ceramide (BC_5_C) upon interaction with the plasma membrane, as BSA dissociates from the complex [30,31,32]. Therefore, in this manuscript, we distinguish between BC_5_C/BSA and BC_5_C depending on the presence or absence of BSA. For quantitative analysis, isolated *Chlorella* cells obtained by either discontinuous Percoll centrifugation or the routine isolation method were kept at 4 °C and an aliquot of this sample was suspended in 5 µM BC_5_C/BSA in placed on glass slides and examined daily after isolation. For each day, multiple slides were prepared and treated as technical replicates, and the proportion of cells retaining PV membranes was determined based on BC_5_C fluorescence labeling. For cells isolated by discontinuous Percoll centrifugation, approximately 110–700 cells were counted per slide, whereas for cells isolated by the routine method, approximately 29–173 cells were counted per slide. The presence of PV membranes was judged by the continuous fluorescence signal surrounding the algal cells.

## 3. Results

### 3.1. Intracellular Distribution of Mitochondria

**Figure 1:**
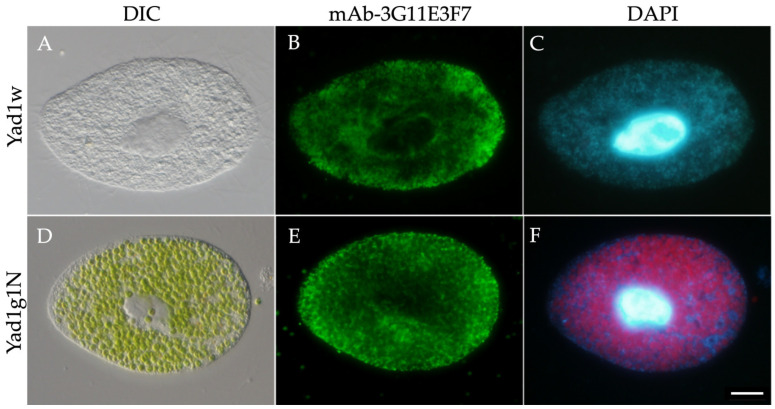
Indirect immunofluorescence staining using the anti-mitochondrial monoclonal antibody mAb-3G11E3F7. (**A**–**C**) Aposymbiotic Yad1w cell. (**D**–**F**) Symbiotic Yad1g1N cell. In Yad1w, mitochondria are dispersed throughout the cytoplasm (**B**). In Yad1g1N, mitochondria are localized in the spaces between *Chlorella* cells (**E**). The left edge: anterior end of the cell. Images were acquired using 10× eyepieces and a 40× objective lens. Scale bar: 20 μm.

To visualize the intracellular distribution of mitochondria, we developed a monoclonal antibody (mAb-3G11E3F7) specific to *P. bursaria* mitochondria and performed indirect immunofluorescence microscopy combined with DAPI staining (Figure 1). Because *P. bursaria* cells were air-dried and fixed on coverslips, they became flattened during preparation (Figure 1A,D). In aposymbiotic Yad1w cells, mitochondria were dispersed throughout the cytoplasm (Figure 1B), whereas in symbiotic Yad1g1N cells, mitochondria were concentrated in the spaces between the endosymbiotic *Chlorella* cells (Figure 1E). Mitochondrial DNA and the host nuclei exhibited DAPI fluorescence (Figure 1C,F). The red signal in panel (Figure 1F) represents chlorophyll autofluorescence from *Chlorella* chloroplasts.

**Figure 2:**
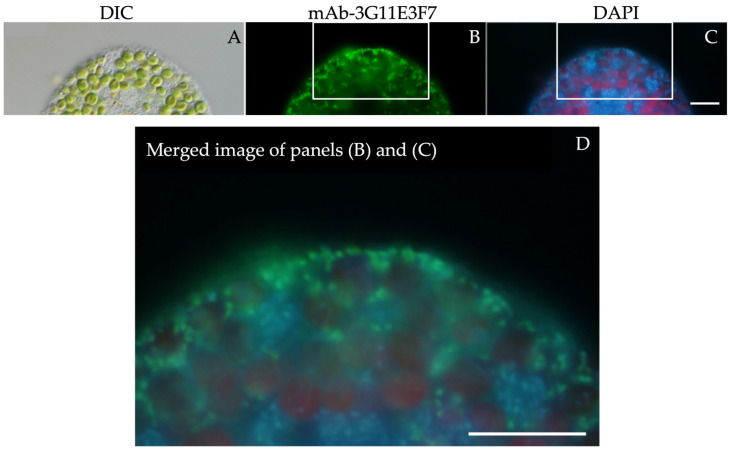
High-magnification indirect immunofluorescence staining of symbiotic Yad1g1N cell using mAb-3G11E3F7 and DAPI. (**A**) DIC image showing endosymbiotic *Chlorella* cells located near the host cell surface. (**B**) Alexa Fluor 488 immunofluorescence showing mitochondria surrounding the endosymbiotic algae. (**C**) DAPI fluorescence showing mitochondrial DNA (light blue) together with chlorophyll autofluorescence from *Chlorella* chloroplasts (red). (**D**) Merged image of the white-lined areas in panels (**B**,**C**). The mitochondrial immunofluorescence signal corresponds well with the mitochondrial DNA signal. Images were acquired using 10× eyepieces and a 100× objective lens. Scale bar: 10 μm.

To confirm the detailed localization of mitochondria in symbiotic Yad1g1N cells, we performed high-magnification observations using a 100× oil-immersion objective (Figure 2). As shown in panel (Figure 2A), endosymbiotic *Chlorella* cells were positioned near the host cell surface and were intimately surrounded by mitochondria (Figure 2B). The particles labeled with mAb-3G11E3F7 were also labeled with DAPI (Figure 2B–D), confirming that the antibody-labeled structures represent mitochondria.

### 3.2. Isolation of Symbiotic Algae Possessing PV Membranes and Mitochondria from Homogenates of Symbiotic P. bursaria

**Figure 3:**
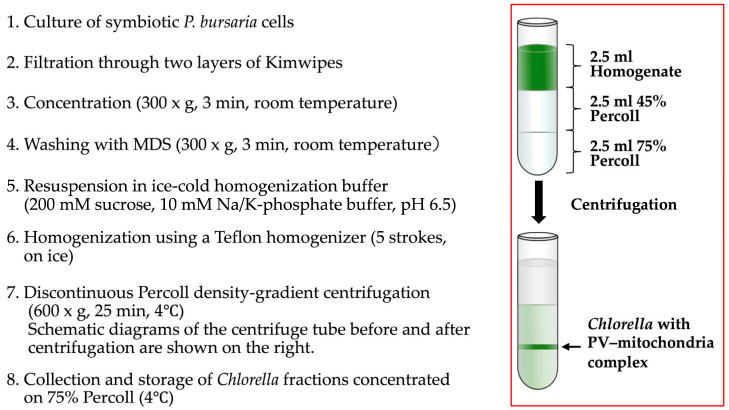
Isolation procedure for symbiotic *Chlorella* retaining PV membranes and associated host mitochondria. Symbiotic *P. bursaria* cells were gently homogenized and subjected to discontinuous Percoll centrifugation. *Chlorella* cells enclosed by PV membranes and associated with host mitochondria were recovered from the interface between Percoll layers.

To determine whether the mitochondria surrounding *Chlorella* cells are firmly attached to the PV membrane rather than merely in close proximity, symbiotic *P. bursaria* cells were gently homogenized, and a *Chlorella*-enriched fraction was obtained using the discontinuous Percoll centrifugation procedure described in Figure 3 and in the Materials and Methods section. After centrifugation, the green *Chlorella*-containing band at the 75%/45% interface was collected with a Pasteur pipette, the concentration of *Chlorella* cells was determined using a hemocytometer, and the sample was stored at 4 °C until use.

**Figure 4:**
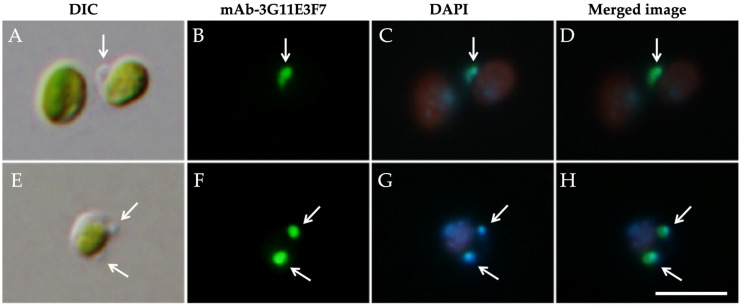
Indirect immunofluorescence staining with mAb-3G11E3F7 and DAPI of symbiotic *C. variabilis* strain 1N cells isolated by discontinuous Percoll centrifugation. (**A**) DIC image showing a small vesicular structure attached to the algal surface (arrow). (**B**) Immunofluorescence labeling with mAb-3G11E3F7. (**C**) DAPI fluorescence showing mitochondrial DNA. (**D**) Merged image confirming that the attached vesicular structure corresponds to host mitochondria. (**E**) DIC image showing two vesicular structures attached to the algal surface (arrows). (**F**) Immunofluorescence labeling with mAb-3G11E3F7 showing two signals. (**G**) DAPI fluorescence showing mitochondrial DNA corresponding to these structures. (**H**) Merged image confirming that these structures correspond to host mitochondria. Red signals (**C**,**G**) represent chlorophyll autofluorescence from *Chlorella* chloroplasts. Images were acquired using 10× eyepieces and a 100× objective lens. Scale bar: 10 μm.

The isolated *Chlorella* fraction was examined by DIC and indirect immunofluorescence microscopy using mAb-3G11E3F7 together with DAPI staining (Figure 4). DIC images revealed small vesicular structures attached to the *Chlorella* cells (Figure 4A,E; white arrows). In panel (Figure 4A), the *Chlorella* cell on the right possessed one vesicle, whereas the cell on the left had none. The cell in panel (Figure 4E) had two vesicles. These vesicles were labeled with mAb-3G11E3F7 (Figure 4B,F) and also exhibited DAPI fluorescence (Figure 4C,D,G,H), demonstrating that the antibody-labeled vesicles correspond to host mitochondria. However, panels (Figure 4B,F) do not allow us to determine whether the antigen recognized by this monoclonal antibody resides on the mitochondrial outer membrane, the inner membrane, or another mitochondrial component. The antigen molecule recognized by this antibody has not yet been identified.

### 3.3. The Attachment Between Endosymbiotic Chlorella and Host Mitochondria Is Mediated by the PV Membrane

**Figure 5:**
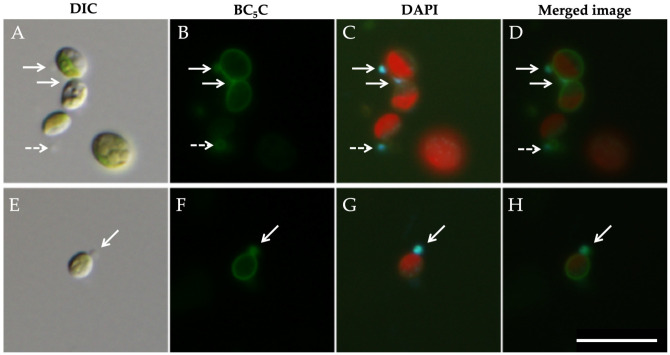
Fluorescence labeling of the PV membrane and host mitochondrial structures using BC_5_C/BSA. Symbiotic *Chlorella* cells isolated by discontinuous Percoll centrifugation were observed by DIC (**A**,**E**), BC_5_C staining (**B**,**F**), and DAPI staining (**C**,**G**). Panels (**D**,**H**) show merged images of BC_5_C and DAPI staining. DIC images show small structures attached to *Chlorella* cells (solid arrows) and one structure not attached (wavy arrow). These structures correspond to mitochondria, as confirmed by DAPI staining. Mitochondria bind only to *Chlorella* cells labeled with BC_5_C and do not bind to unlabeled cells. BC_5_C labels the outer surface of the algae (**B**,**F**). Because the PV membrane is the only known structure surrounding the outside of the *Chlorella* cell wall, these observations suggest that adhesion between *Chlorella* and mitochondria is mediated by interactions involving the PV membrane and mitochondrial structures. The red signal (**C**,**G**) represents chlorophyll autofluorescence from *Chlorella* chloroplasts. Images were acquired using 10× eyepieces and a 100× objective lens. Scale bar: 10 μm.

Incubating the *Chlorella* fraction isolated by discontinuous Percoll centrifugation (Figure 3) with 5 µM BC_5_C/BSA for 30 min at room temperature in the dark enabled fluorescent labeling of both the PV membrane surrounding the endosymbiotic *Chlorella* cell and the mitochondrial membrane. Although the PV membrane has previously been observed by electron microscopy, this study provides the first fluorescent visualization of the PV membrane under light microscopy. BC_5_C/BSA is widely used as a fluorescent probe for labeling the Golgi apparatus and tracing sphingolipid trafficking in eukaryotic cells [30,31,32]. Although typically used for this purpose, we found—as expected—that BC_5_C labels the PV membrane and mitochondrial structures, enabling fluorescent visualization of these membranes (Figure 5).

As described in Figure 5 legend, BC_5_C labeling revealed that mitochondria associate specifically with PV membrane-enclosed *Chlorella* cells and not with unlabeled cells. Because the distance between the PV membrane and the *Chlorella* cell wall is very small [19,20,21], the presence or absence of the PV membrane cannot be determined from DIC images alone (Figure 5A,E). However, BC_5_C clearly labeled the outer surface of the algae (Figure 5B,F), indicating that BC_5_C is localized to the PV membrane and associated mitochondrial structures. These observations consistently suggest that adhesion between *Chlorella* and mitochondria involves interactions between the PV membrane and mitochondrial structures. Furthermore, the mitochondrion indicated by the wavy arrow in panel (Figure 5B) exhibited ring-shaped fluorescence, consistent with BC_5_C labeling of mitochondrial structures.

BC_5_C/BSA contains BSA, which has a molecular weight of approximately 66 kDa and a molecular diameter of approximately 7 nm in its unbound form [33]. The pore size of plant and algal cell walls is typically approximately 3–5 nm [34], indicating that BSA is unlikely to pass through these pores. Molecules of this size are also generally unable to permeate the plasma membrane [35]. However, the interaction between BC_5_C and BSA is weak. When BC_5_C/BSA approaches the plasma membrane, BC_5_C likely dissociates from BSA because of its higher affinity for the hydrophobic core of the membrane and subsequently associates with the membrane. The fluorescent labeling observed in panels (Figure 5B,F) indicates that BC_5_C is localized to the PV membrane and the mitochondrial structures. These observations suggest that adhesion between *Chlorella* and mitochondria is maintained through the association between the PV membrane and the mitochondrial structures. In contrast, *Chlorella* cells that were not labeled with BC_5_C retained normal morphology, indicating that BC_5_C associates with the PV membrane but not with the *Chlorella* cell wall. If BC_5_C/BSA or BC_5_C were able to penetrate the cell wall, labeling of the *Chlorella* plasma membrane and other internal membranes would be expected; however, such labeling was not observed.

Up to three mitochondria were observed to attach to a single PV-enclosed *Chlorella* cell. The two *Chlorella* cells shown at the top of panels (Figure 5A–D) appear to be connected by a single mitochondrion that attaches to the PV membranes of both cells. However, it remains unclear whether this represents two PV-enclosed *Chlorella* cells, each bearing one mitochondrion and positioned in close proximity, or a single mitochondrion attached to a PV-enclosed *Chlorella* cell undergoing binary fission. The red signal (Figure 5C,G) represents chlorophyll autofluorescence from *Chlorella* chloroplasts.

### 3.4. Stability of PV Membranes After Isolation by Discontinuous Percoll Density Gradient Centrifugation

**Figure 6:**
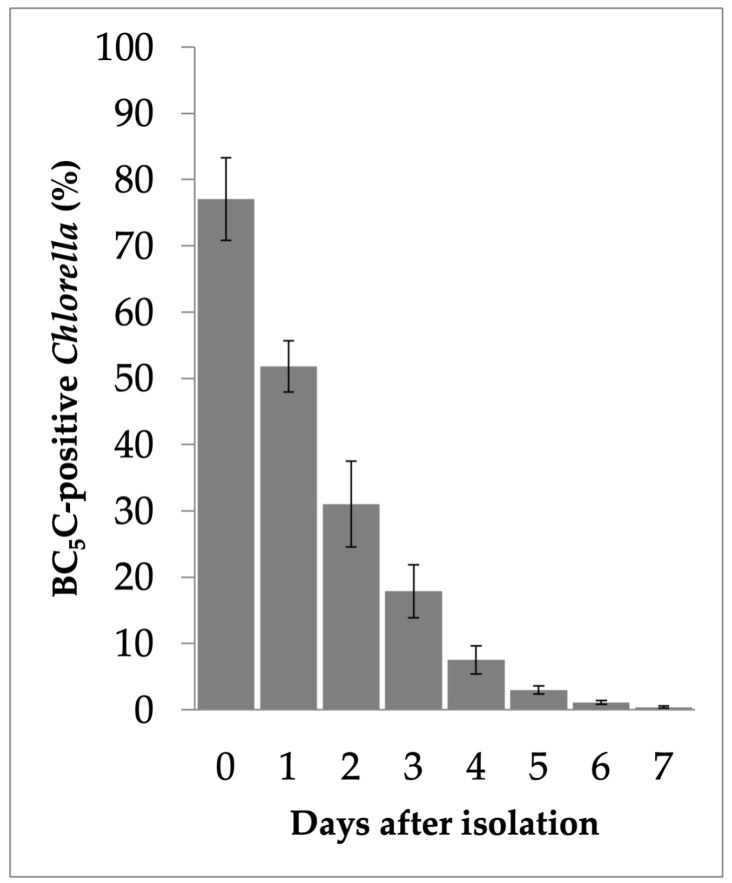
Time-dependent loss of PV membranes in *Chlorella* cells isolated by discontinuous Percoll centrifugation. The isolated *Chlorella* fraction was stored at 4 °C, and the proportion of cells retaining PV membranes was assessed daily by BC_5_C fluorescence labeling. Each data point represents the mean of technical replicates (*n* = 8–10 slides), with approximately 110–700 cells counted per slide. The mean percentage of BC_5_C-positive *Chlorella* and the corresponding 95% CI were calculated. The percentage of PV-retaining cells decreased progressively over time. Error bars represent 95% Confidence intervals (CI).

To quantify the stability of PV membranes after isolation, the *Chlorella* fraction obtained by discontinuous Percoll centrifugation was incubated with BC_5_C/BSA from day 0 to day 7. At day 0, 77.1 ± 6.2% (95% CI) of isolated algae retained PV membranes. This proportion decreased to 51.8 ± 3.9% on day 1, 31.0 ± 6.5% on day 2, 17.9 ± 4.0% on day 3, 7.5 ± 2.1% on day 4, 3.0 ± 0.6% on day 5, 1.1 ± 0.3% on day 6, and 0.4 ± 0.2% on day 7 (Figure 6).

**Figure 7:**
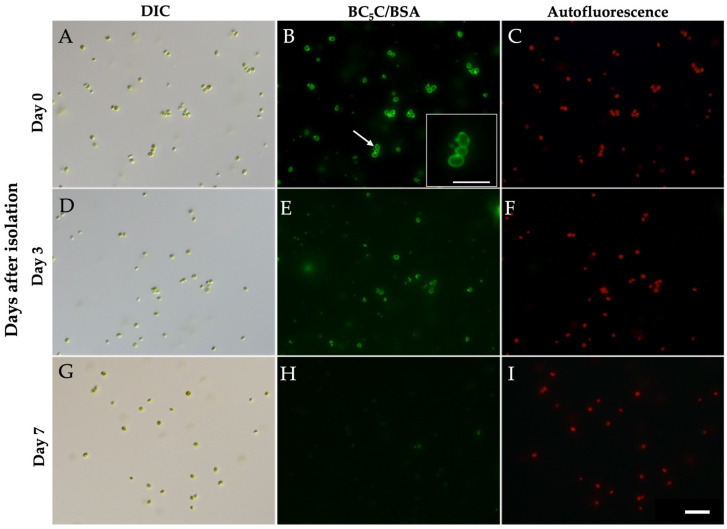
Representative BC_5_C fluorescence images showing progressive loss of PV membranes after isolation by discontinuous Percoll centrifugation. (**A**) DIC image at Day 0. (**B**) BC_5_C fluorescence image at Day 0 showing PV membrane labeling; bright fluorescent puncta correspond to associated mitochondria. (**C**) Chlorophyll autofluorescence at Day 0. (**D**) DIC image at Day 3 after isolation. (**E**) BC_5_C fluorescence image at Day 3 showing reduced fluorescence intensity and fewer PV-positive cells. (**F**) Chlorophyll autofluorescence at Day 3. (**G**) DIC image at Day 7 after isolation. (**H**) BC_5_C fluorescence image at Day 7 showing further loss of fluorescence signals. (**I**) Chlorophyll autofluorescence at Day 7. An enlarged view of the three-Chlorella cell cluster indicated by an arrow in panel (**B**) is shown in the lower-right corner. Images were acquired using 10× eyepieces and a 40× objective lens. Scale bars: 10 μm in (**I**) and 20 μm in (**B**). Panels (**C**,**F**,**I**) show chlorophyll autofluorescence. Images were acquired using 10× eyepieces and a 40× objective lens. Scale bars: 10 µm in (**I**) and 20 µm in (**B**).

Representative BC_5_C labeling images from days 0, 3, and 7 are shown in Figure 7. By day 3, both the number of BC_5_C-labeled algae and the fluorescence intensity had decreased markedly (Figure 7D–F). By day 7, BC_5_C-labeled *Chlorella* cells had almost completely disappeared (Figure 7G–I). In panel (Figure 7B), several small, strongly fluorescent dots are visible on the BC_5_C-labeled PV membrane. These correspond to the mitochondria observed in Figure 5B,F. From panels (Figure 7A,B), 65% of BC_5_C-positive *Chlorella* cells had attached mitochondria. A cluster consisting of three to four *Chlorella* cells is present, and enlarged views of the three-cell cluster indicated by an arrow in panel (Figure 7B) are shown in the lower right corner. Three mitochondria are attached to the PV membrane of the uppermost *Chlorella* cell in this cluster. It remains unclear whether this cluster formed within the cytoplasm of *P. bursaria*, during isolation of the *Chlorella* fraction, or during preparation of the microscopic specimen.

**Figure 8:**
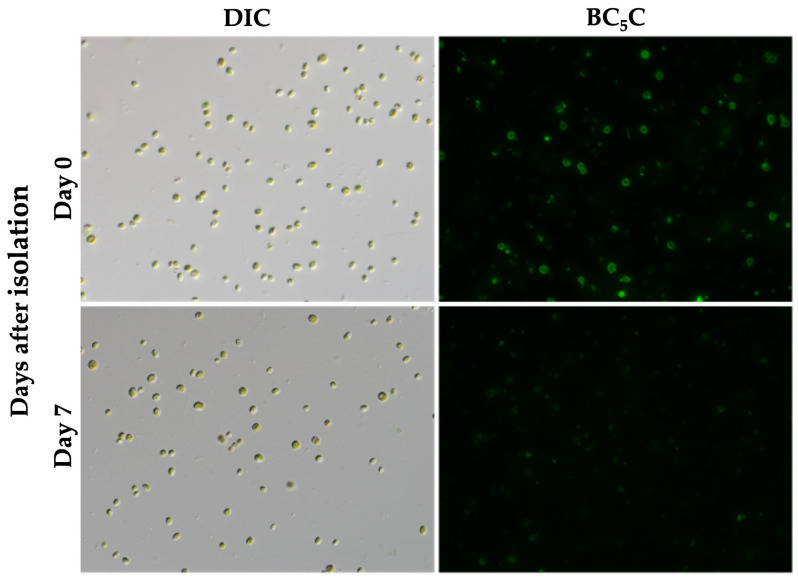
Reduced retention of PV membranes in *Chlorella* cells isolated without Percoll centrifugation. Symbiotic *P. bursaria* cells were isolated using routine isolation method as shown in Materials and labeled with BC_5_C. At day 0, approximately 11.1 ± 2.3% (95% CI) of cells retained PV membranes. Each data point represents the mean of technical replicates (*n* = 20 slides), with approximately 95–173 cells counted per slide. By day 7, PV membrane labeling was rarely observed. Images were acquired using 10× eyepieces and a 40× objective lens. Scale bar: 20 μm.

Figure 8 shows BC_5_C labeling images at days 0 and 7 after isolation of symbiotic *C. variabilis* strain 1N cells using the routine isolation method without discontinuous Percoll centrifugation. At day 0, approximately 11.1 ± 2.3% (95% CI) of the cells retained PV membranes, based on technical replicates (*n* = 20 slides) with approximately 95–173 cells counted per slide. BC_5_C-labeled *Chlorella* cells had almost completely disappeared by day 7. BC_5_C-labeled *Chlorella* cells at day 1 were 0.1 ± 0.1% (CI) of the cells (see Supplementary Materials, Quantitative_Data for Figure 6, D1). Compared with cells isolated using discontinuous Percoll centrifugation, both the proportion of BC_5_C-labeled cells and the proportion of PVs with attached mitochondria were lower.

The decrease in the proportion of PV membrane-bearing *Chlorella* after isolation by discontinuous Percoll centrifugation or by the routine isolation method was reproducibly observed in multiple independent experiments using BC_5_C staining, although the corresponding data were not recorded.

## 4. Discussion

### 4.1. Direct Evidence for a Structurally Stable PV–Mitochondrial Adhesion

The present study provides direct evidence that the perialgal vacuole (PV) membrane surrounding endosymbiotic *Chlorella* in *Paramecium bursaria* forms a structurally stable physical association with the host mitochondrial structures. By combining discontinuous Percoll centrifugation, fluorescent labeling with BC_5_C/BSA, and a mitochondria-specific monoclonal antibody, we demonstrate that mitochondria remain attached to PV-enclosed *Chlorella* even after mechanical disruption by homogenization and subsequent centrifugation. This persistence indicates that the PV–mitochondrial interaction is mechanically robust rather than a transient or incidental contact. Previous TEM studies have consistently shown close apposition between the PV membrane and mitochondrial outer membrane [19,20,21], often described as a “cage-like” arrangement surrounding the alga [21], suggesting a specialized architecture for metabolic exchange. However, TEM alone cannot distinguish between mere proximity and physical adhesion. The present findings bridge this gap by demonstrating that *Chlorella* cells frequently retain associated mitochondria after isolation and that these components co-sediment during centrifugation. Together, these observations provide definitive structural evidence that the PV membrane and mitochondria are tightly associated.

Comparable membrane–mitochondrion associations have been reported in parasitic systems such as *Toxoplasma gondii*, in which the parasitophorous vacuole membrane forms a high-affinity interaction with host mitochondria mediated by parasite-derived factors [36,37], and parasitophorous vacuole membrane serves as a critical interface for host–parasite interactions [38]. These studies support the concept that stable membrane–mitochondrial interactions can be established across diverse intracellular systems.

### 4.2. Fluorescent Visualization of the PV Membrane Using BC_5_C/BSA

A key advance of this study is the successful fluorescent visualization of the PV membrane using BC_5_C/BSA. Although BC_5_C/BSA is widely used to label the Golgi apparatus and trace sphingolipid trafficking in eukaryotic cells [31,39], its application to the PV membrane and mitochondrial membrane in isolated symbiotic units has not previously been reported.

In this study, BC_5_C/BSA enabled simultaneous visualization of both the PV membrane and the mitochondrial membrane by light microscopy. The labeling mechanism is consistent with the known membrane-insertion behavior of BC_5_C: upon approaching cellular membranes, BC_5_C likely dissociates from BSA and partitions into lipid bilayers owing to its high affinity for hydrophobic membrane environments. This property allows BC_5_C to label both the PV membrane and the mitochondrial membrane. Iwamoto and Allen [40] demonstrated in *P. multimicronucleatum* (*Paramecium* species unable to maintain symbiotic algae) that BC_5_C is internalized from the plasma membrane into the cytoplasm in an ATP-dependent manner, and that depletion of ATP causes BC_5_C to accumulate in the plasma membrane. This finding is consistent with our interpretation that BC_5_C incorporated into the PV membrane surrounding isolated *Chlorella* cells remains in the membrane because the isolated organelles lack the intracellular transport machinery required for further trafficking of the probe.

Importantly, no fluorescence was detected within the *Chlorella* cell interior, supporting the interpretation that BC_5_C does not penetrate the algal cell wall but instead selectively labels the most external plasma membrane of isolated organelles. This approach therefore represents, to our knowledge, the first optical method for visualizing the PV membrane, which has previously been accessible only by electron microscopy [19,20,21]. The ability to observe PV membranes in isolated symbiotic units provides a powerful tool for future analyses of host–symbiont membrane interactions.

### 4.3. Mechanistic Implications of PV–Mitochondrial Adhesion

The demonstration of a structurally stable PV–mitochondrial association raises important questions regarding the underlying molecular mechanisms. One possibility is that the adhesion is mediated by specific protein complexes that bridge the PV membrane and the mitochondrial outer membrane, analogous to membrane contact site proteins described in other eukaryotic systems [25,26]. Alternatively, lipid-based interactions, such as microdomain (lipid raft)-like structures [41,42], may contribute to the stabilization of membrane–membrane contact. Because BC_5_C labeling indicates that the interaction occurs at the level of the mitochondrial membrane, the adhesion most likely involves direct membrane–membrane association rather than cytoskeletal anchoring alone. Up to now, all TEM observations indicate that the two membranes are in close apposition, without an obvious involvement of cytoskeletal elements as intermediaries [19,20,21]. The observation that mitochondria remain attached to PV membranes after homogenization further suggests that the interaction is sufficiently strong to resist mechanical disruption, consistent with a structurally reinforced interface. Functionally, such stable adhesion may contribute to the precise positioning of endosymbiotic *Chlorella* beneath the host cortex, thereby facilitating efficient metabolic exchange, including the transfer of photosynthetically derived metabolites and oxygen [7,8,9]. The maintenance of this spatial organization is therefore likely critical for the stability of the symbiotic relationship [16,20].

Future studies aimed at identifying the molecular components of this interface—such as proteomic analyses of isolated PV–mitochondrial complexes, cryo-electron tomography, identification of the antigen recognized by the mitochondria-specific monoclonal antibody, and the development of monoclonal antibodies specific to the PV membrane or the PV–mitochondrial contact site, followed by antigen identification—will be essential to elucidate the mechanistic basis of this adhesion. We have previously analyzed changes in gene expression in the host *P. bursaria* before and after the establishment of endosymbiosis using transcriptome analysis [4]. Complementary transcriptomic analyses of the symbiotic *Chlorella* will therefore be required to achieve a more comprehensive understanding of this interaction including efficient material exchanges.

### 4.4. Comparative and Evolutionary Perspectives Across Photosynthetic Symbioses

In various photosynthetic symbioses, intracellular phototrophs are enclosed within host-derived membranes that are structurally analogous to the PV membrane. In systems such as *Hydra viridissima* [43,44,45], *Stentor pyriformis* [46,47], and *Mayorella* [48,49], symbiotic algae are similarly surrounded by host membranes that function as regulated interfaces for metabolite exchange. In cnidarian–dinoflagellate symbioses (e.g., corals), the symbiosome membrane has been shown to contain transporters and proton pumps that regulate the microenvironment of the symbiont [24]. These host-derived membranes are generally interpreted as dynamic interfaces that control nutrient flux, pH, and symbiont density, rather than as passive remnants of phagocytosis. However, in most of these systems, the structural relationship between the symbiosome membrane and host organelles has not been resolved in terms of mechanical stability.

In this context, the present study provides the first direct evidence that a host-derived symbiotic interface can establish a mechanically stable adhesion with host mitochondria, thereby extending the functional concept of symbiosome-like membranes to include structural integration with host organelles. These comparisons suggest that the PV membrane in *P. bursaria* represents a specialized and potentially conserved interface that integrates both structural and metabolic functions in photosynthetic endosymbiosis.

### 4.5. Relationship to Previous Physiological Evidence

Previous physiological studies have provided indirect evidence for dynamic interactions between PV-enclosed *Chlorella* and host cellular structures [19,20,21]. In particular, centrifugation experiments on living *P. bursaria* cells demonstrated that endosymbiotic algae detach from their subcortical positions and accumulate at the posterior end of the cell under centrifugal rapidly return to their original positions once the force is removed [22]. These observations imply the existence of an active mechanism that maintains and restores the spatial association of symbiotic algae. However, these earlier studies did not resolve the structural basis of this interaction. The present work complements these physiological observations by demonstrating that the PV membrane and host mitochondria remain physically associated even after mechanical disruption and isolation procedures.

Thus, while previous studies suggested the presence of a dynamic and regulated attachment system, the current findings provide the missing structural evidence, establishing that the PV–mitochondrial interaction represents a mechanically stable membrane–membrane adhesion.

## 5. Conclusions

This study demonstrates that the PV membrane and the host mitochondrial structures form a stable physical association that persists even after homogenization and centrifugation. As expected from the membrane-insertion properties of BC_5_C, fluorescent labeling enabled direct visualization of both membranes under light microscopy, providing, to our knowledge, the first optical method for observing the PV membrane and mitochondria surrounding endosymbiotic *Chlorella*. These findings establish a mechanistic basis for the stable positioning of endosymbionts beneath the host cortex and introduce a new experimental approach for studying membrane interactions in photosynthetic symbioses.

## Data Availability

The raw data supporting the findings of this study are provided as Supplementary Materials. Additional data are available from the corresponding author upon reasonable request.

## Supplementary Materials

The following supporting information can be downloaded at: https://www.mdpi.com/article/10.3390/biom16040561/s1, raw fluorescence images corresponding to Figure 1, Figure 2, Figure 3, Figure 4, Figure 5, Figure 7 and Figure 8, quantitative datasets for Figure 6 used for statistical analyses, and a README file describing the contents.

## Abbreviations

The following abbreviations are used in this manuscript:

PV	Perialgal Vacuole
BC_5_C/BSA	BODIPY FL C_5_-ceramide complexed to BSA
BC_5_C	BODIPY FL C_5_-ceramide
TEM	Transmission Electron Microscopy
DV	Digestive Vacuole
MDS	Modified Dryl’s Solution
SIP	Stock Isotonic Percoll
PBS	Phosphate-Buffered Saline
PBST	PBS-Containing 0.05% (*v*/*v*) Tween 20
DAPI	4′,6-DiAmidino-2-PhenolIndole
DIC	Differential Interference Contrast
BSA	Bovine Serum Albumin
Cl	Confidence Intervals
