# Supplementary figures and images for: First Direct Evidence for a Structurally Stable Adhesion Between the Perialgal Vacuole Membrane and Host Mitochondria in the Paramecium-Chlorella Endosymbiosis
Source: Biomolecules. 2026 Apr 10;16(4):561. doi: 10.3390/biom16040561 (PMC13115306; doi:10.3390/biom16040561)

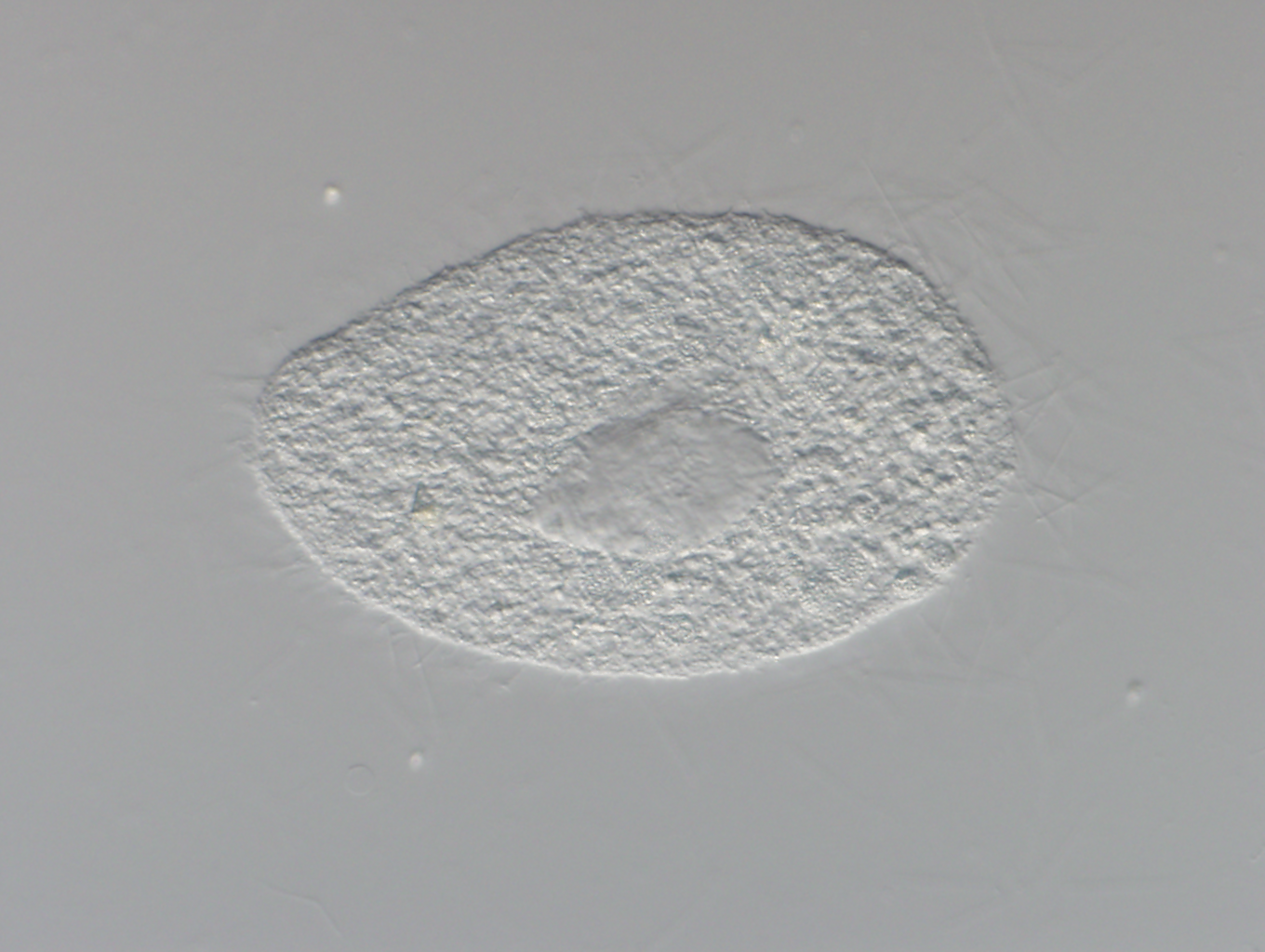

Supplement: Supplementary file 1 [file biomolecules-16-00561-s001.zip › Raw_images Fig1-5,7,8/Fig1A_raw.tif]

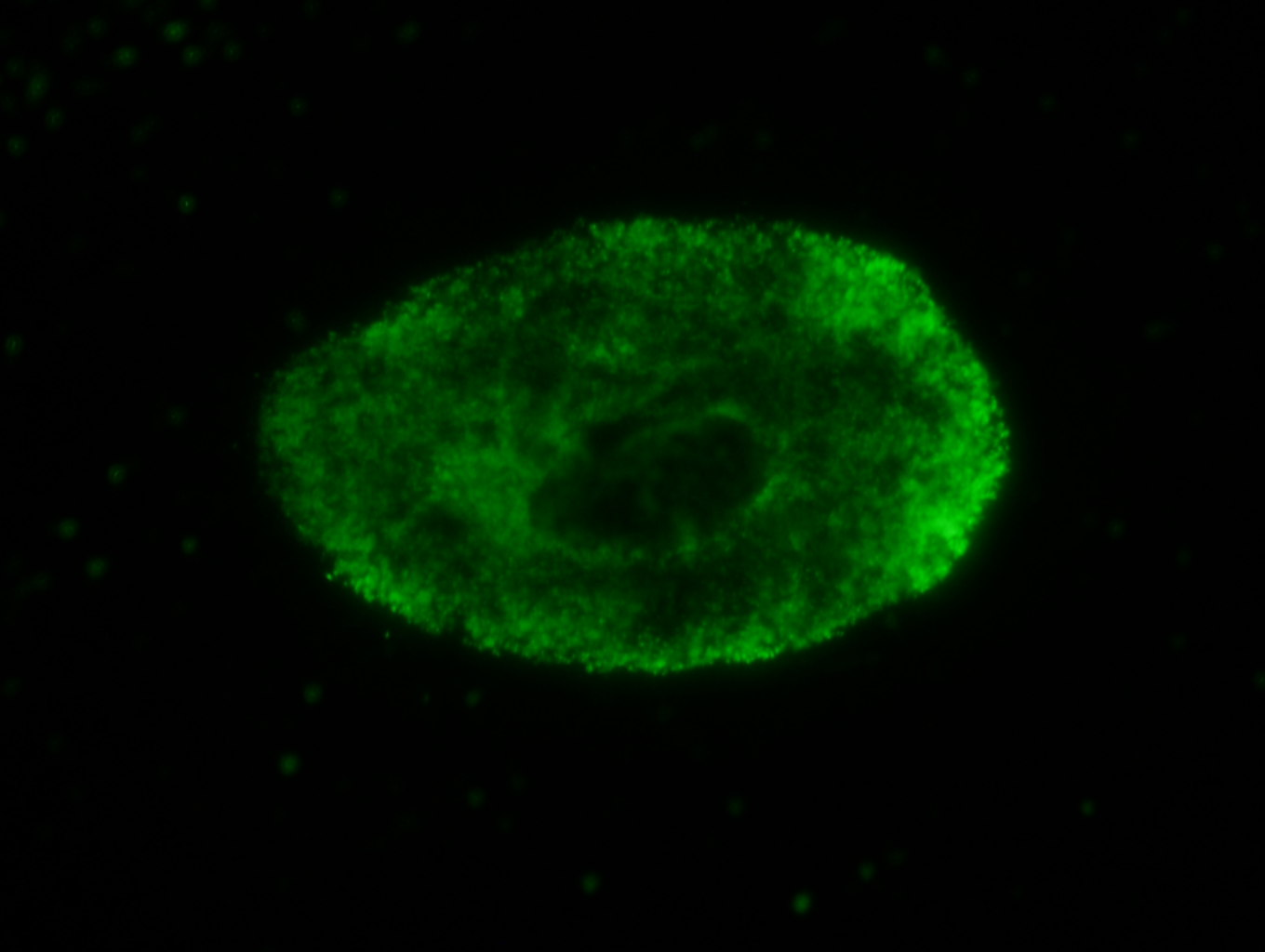

Supplement: Supplementary file 1 [file biomolecules-16-00561-s001.zip › Raw_images Fig1-5,7,8/Fig1B_raw.tif]

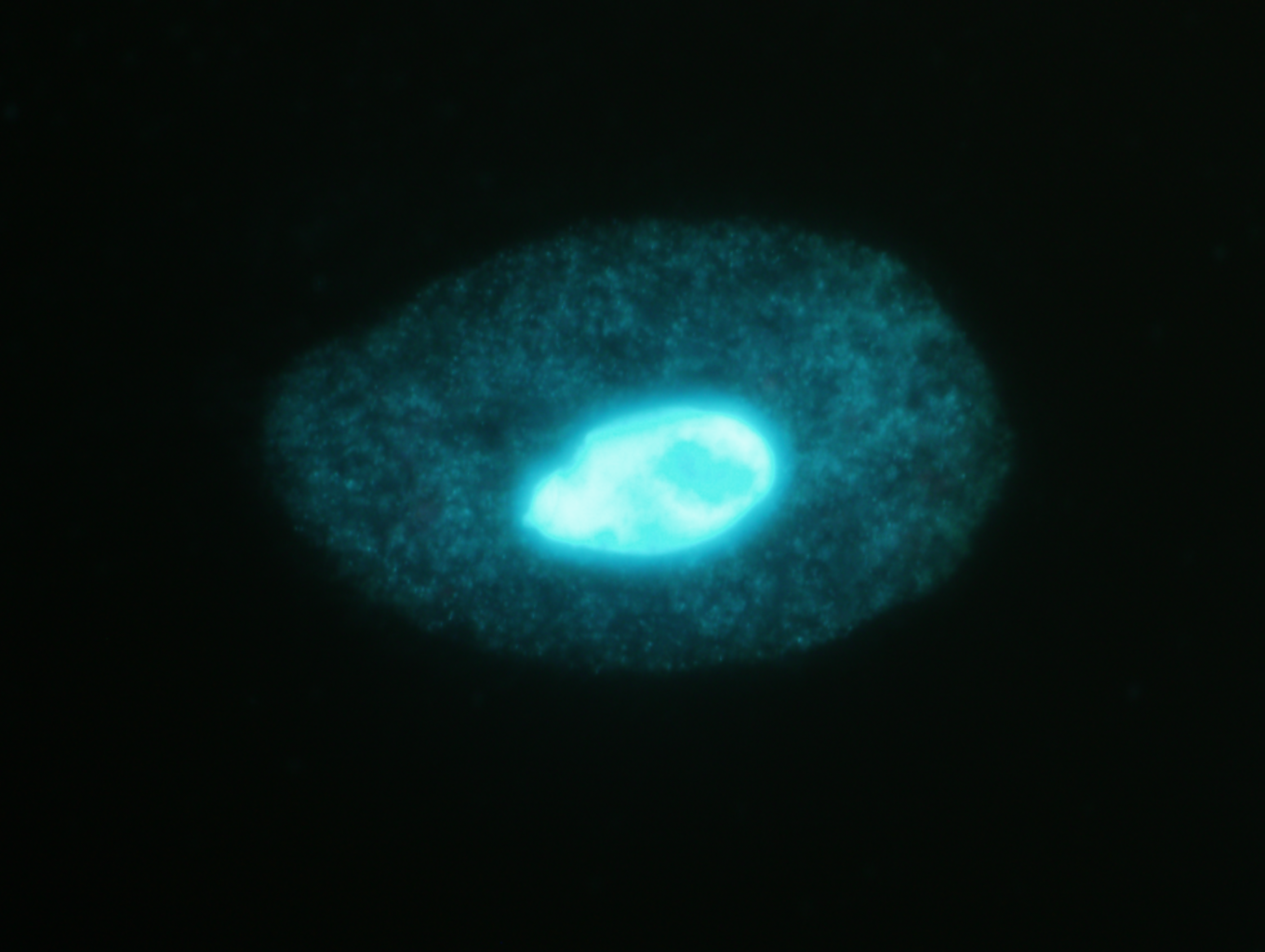

Supplement: Supplementary file 1 [file biomolecules-16-00561-s001.zip › Raw_images Fig1-5,7,8/Fig1C_raw.tif]

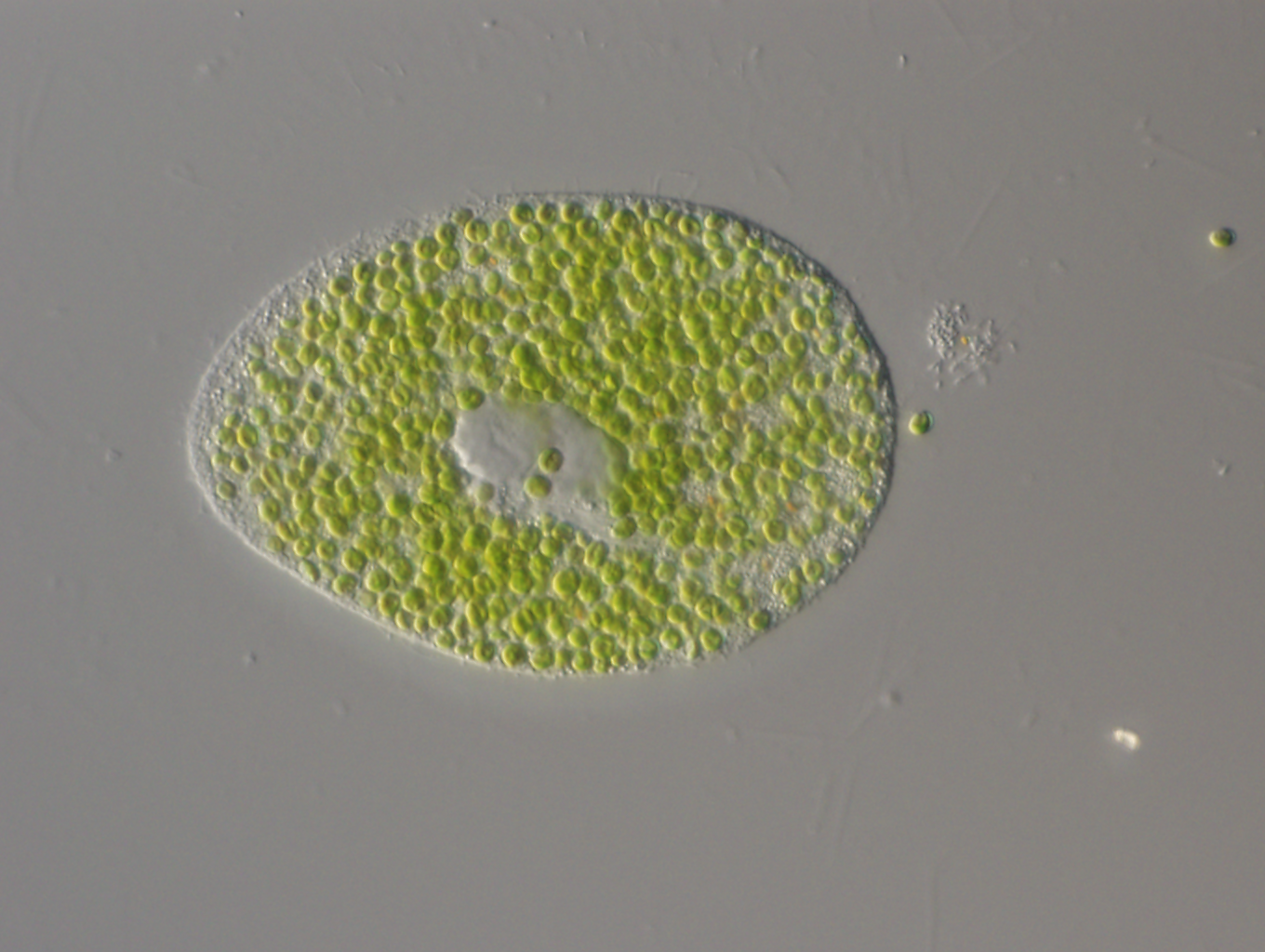

Supplement: Supplementary file 1 [file biomolecules-16-00561-s001.zip › Raw_images Fig1-5,7,8/Fig1D_raw.tif]

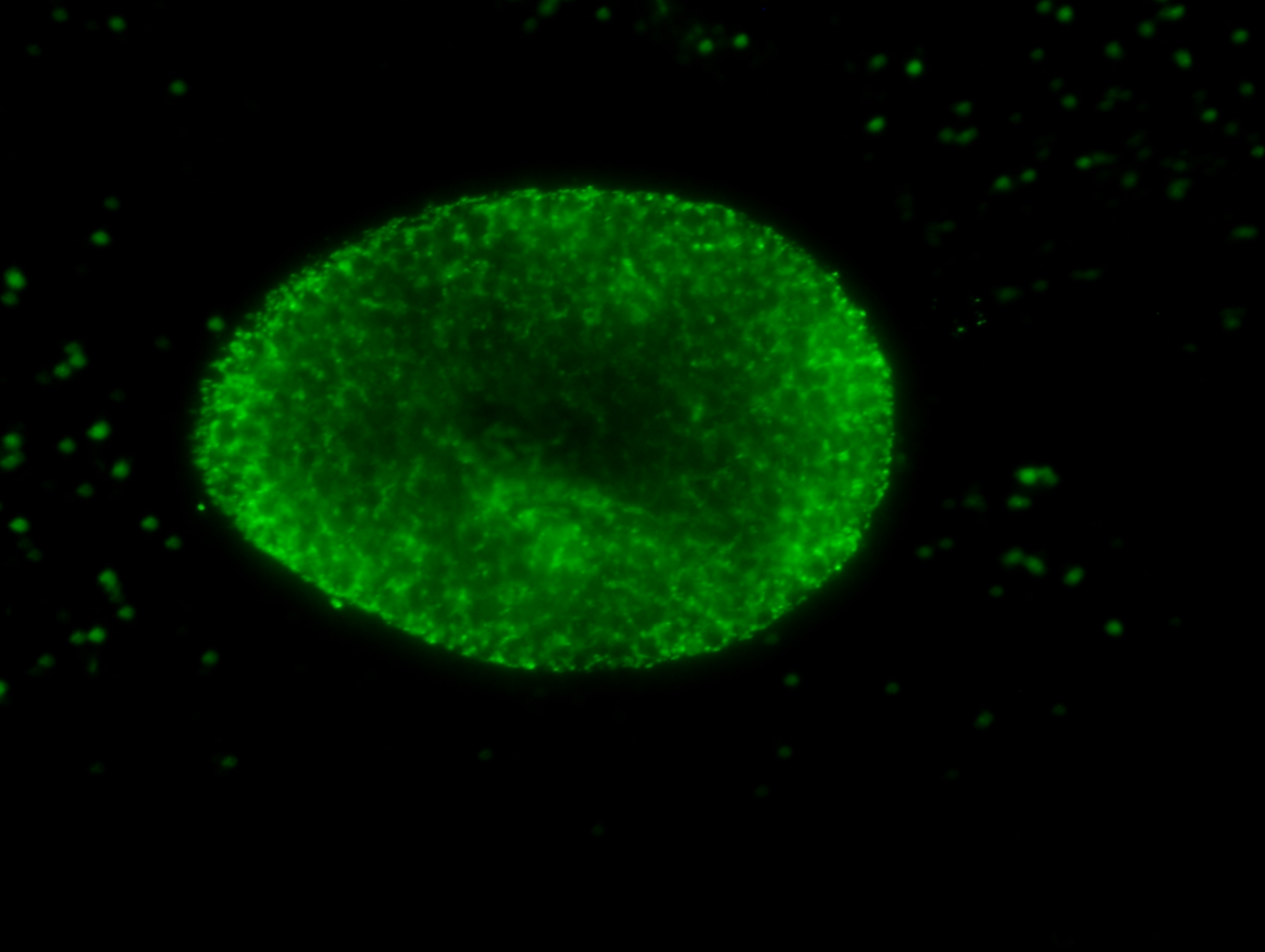

Supplement: Supplementary file 1 [file biomolecules-16-00561-s001.zip › Raw_images Fig1-5,7,8/Fig1E_raw.tif]

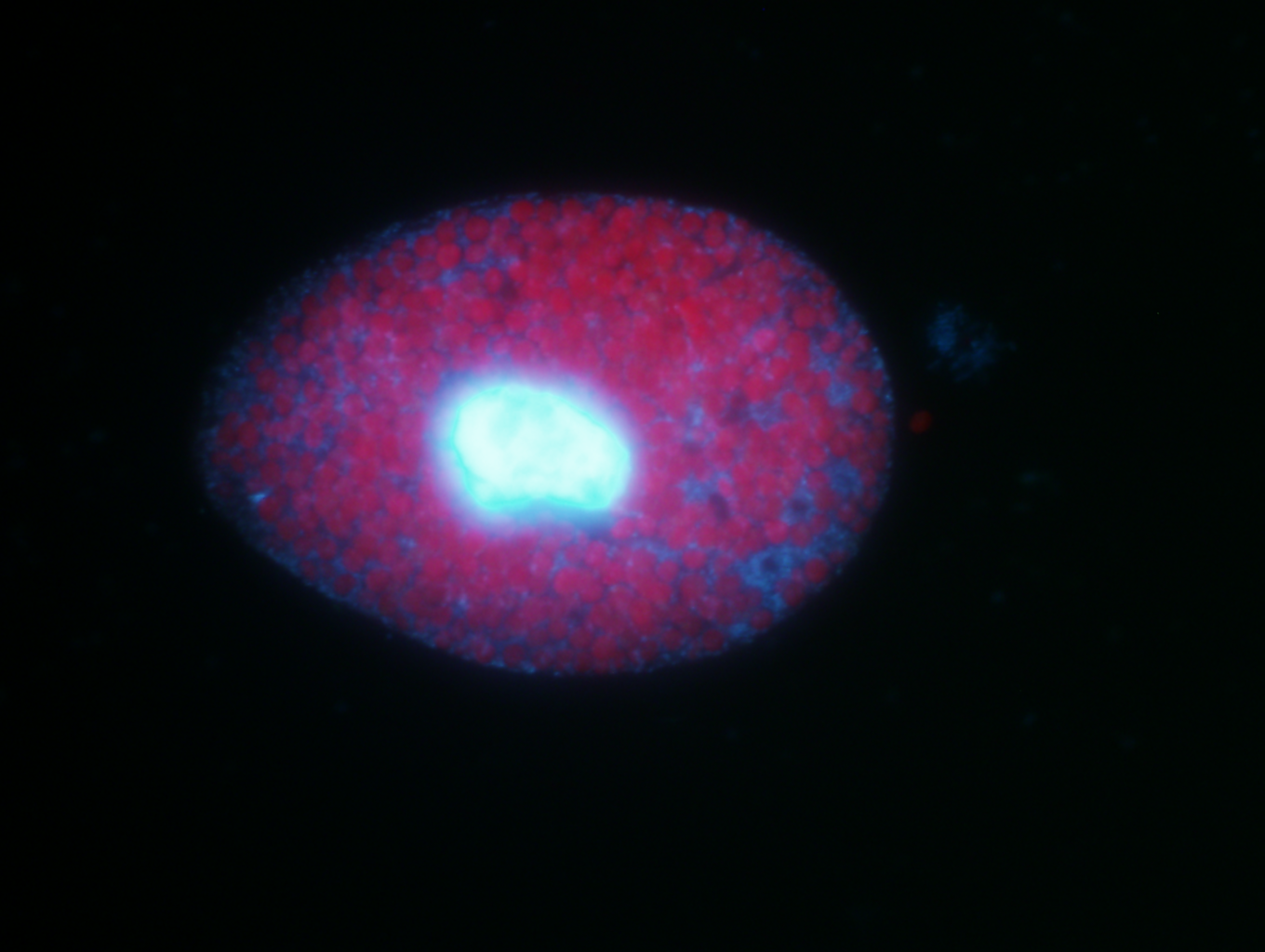

Supplement: Supplementary file 1 [file biomolecules-16-00561-s001.zip › Raw_images Fig1-5,7,8/Fig1F_raw.tif]

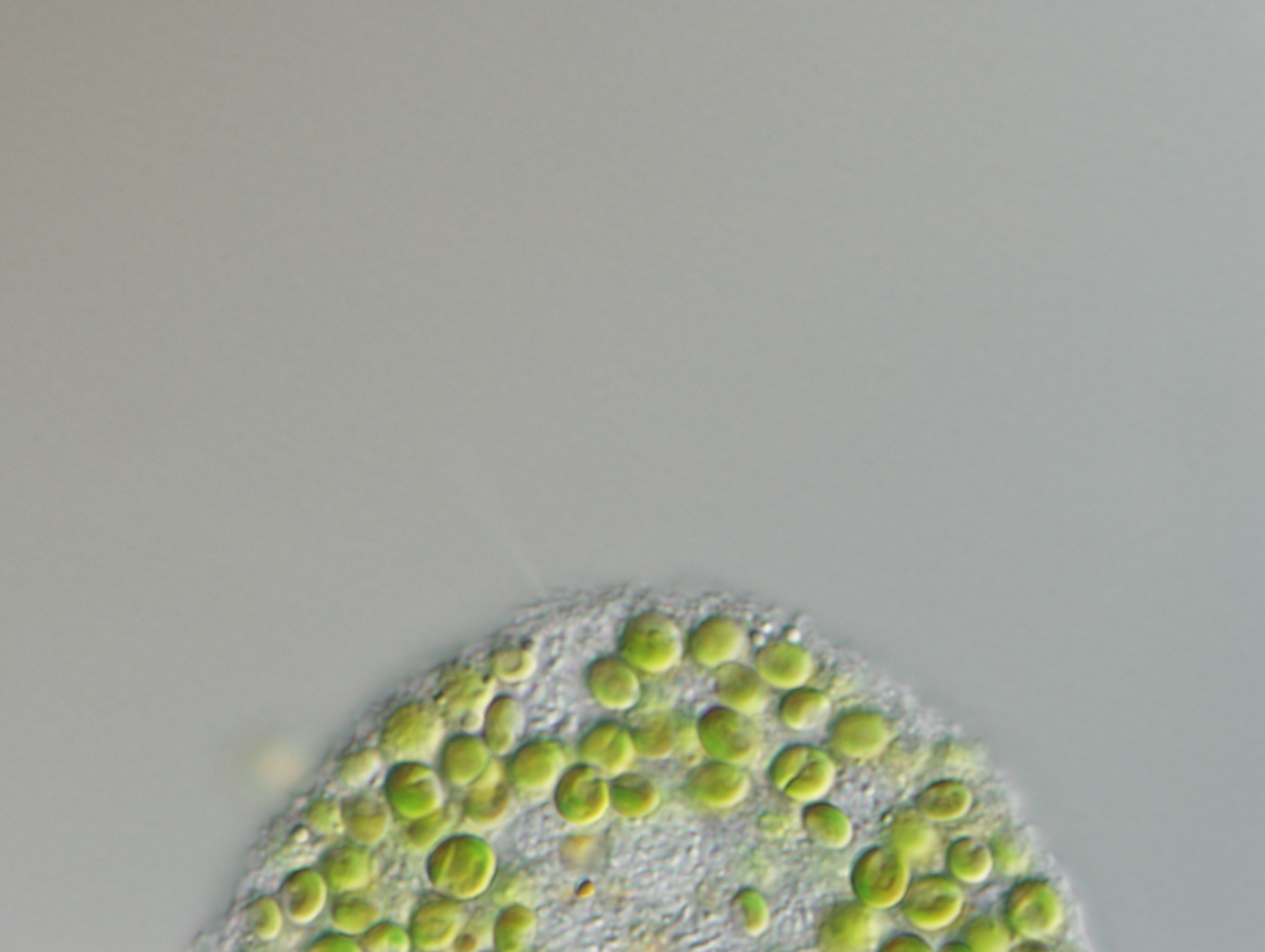

Supplement: Supplementary file 1 [file biomolecules-16-00561-s001.zip › Raw_images Fig1-5,7,8/Fig2A_raw.tif]

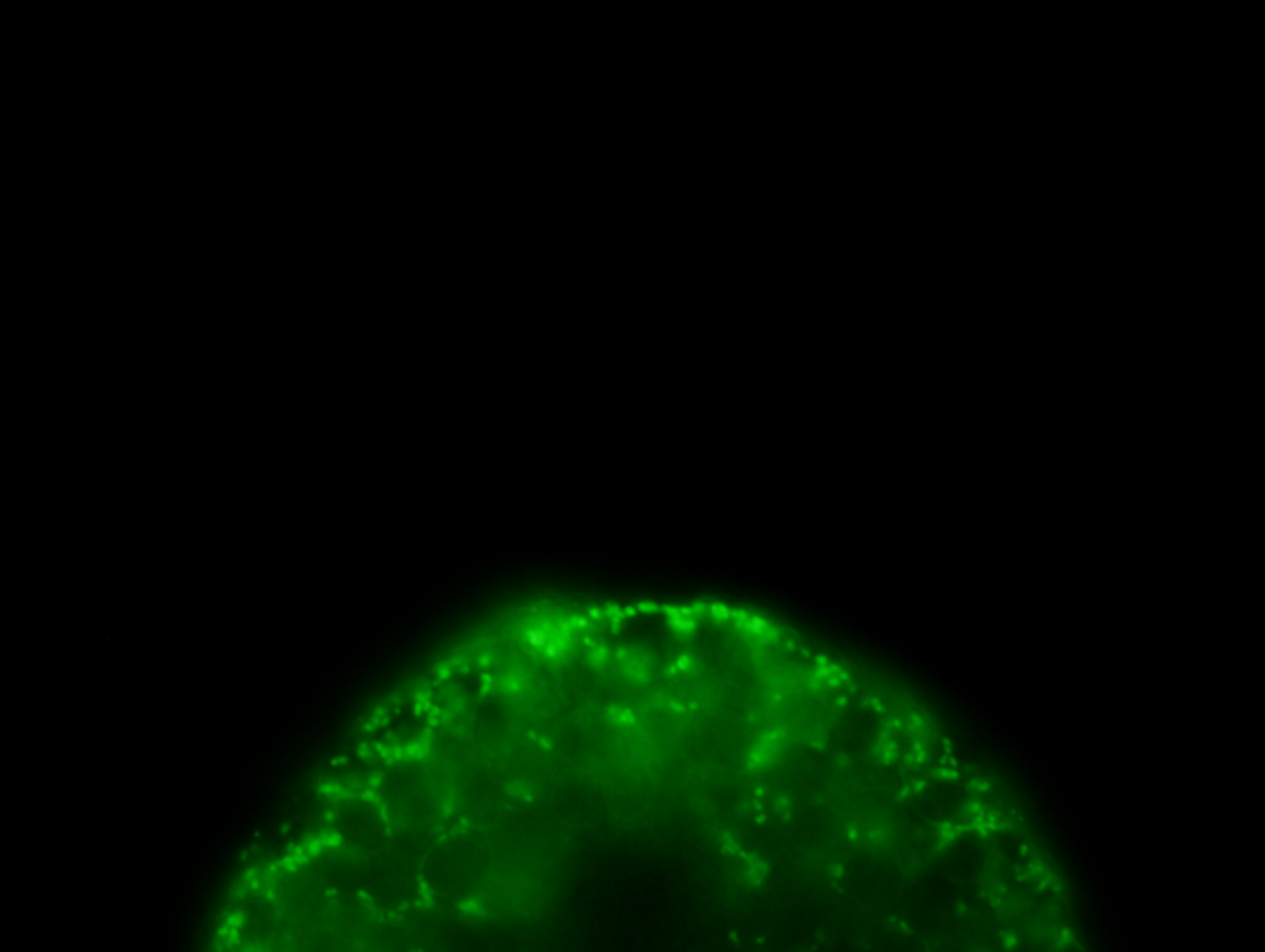

Supplement: Supplementary file 1 [file biomolecules-16-00561-s001.zip › Raw_images Fig1-5,7,8/Fig2B_raw.tif]

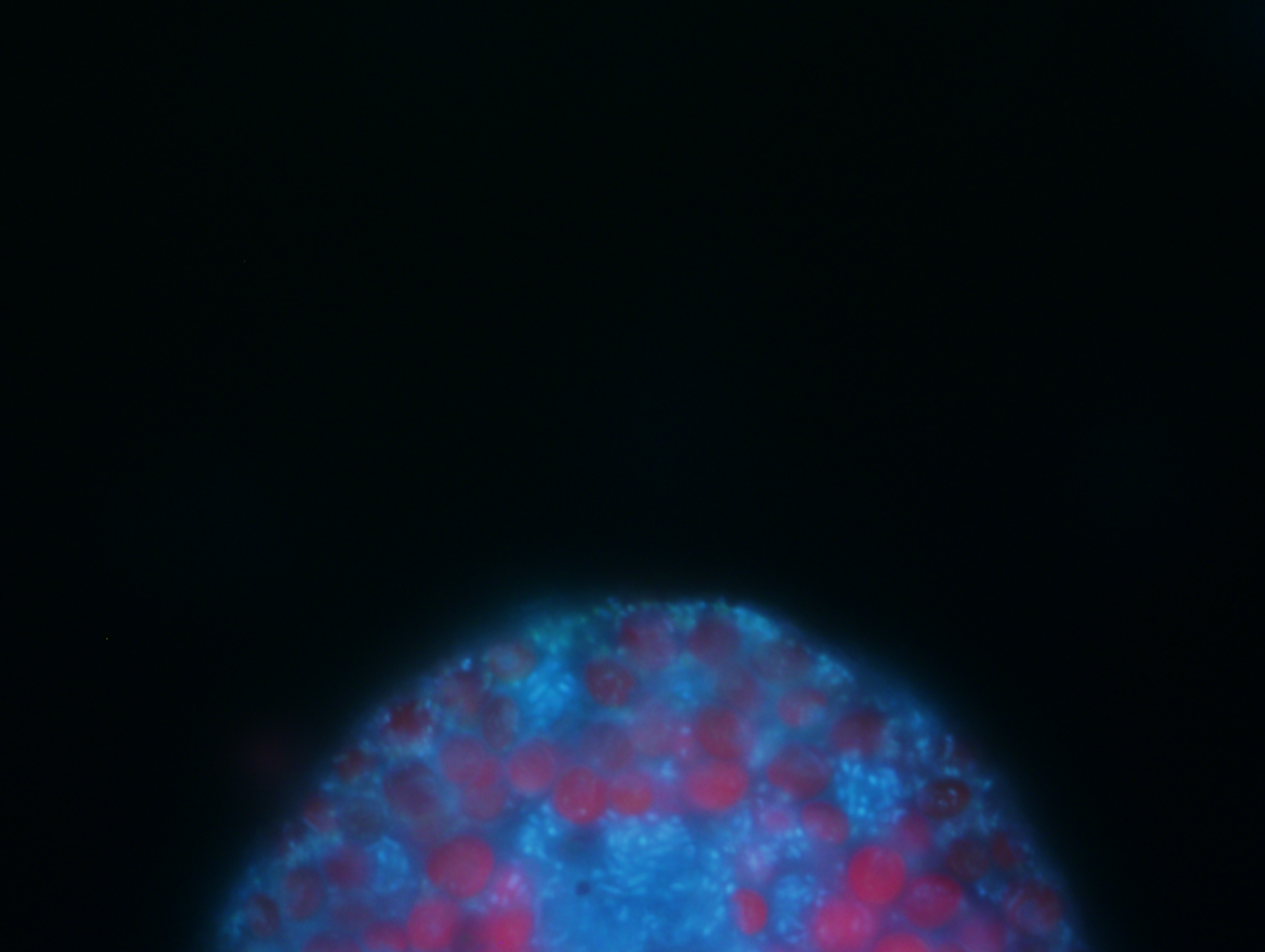

Supplement: Supplementary file 1 [file biomolecules-16-00561-s001.zip › Raw_images Fig1-5,7,8/Fig2C_raw.tif]

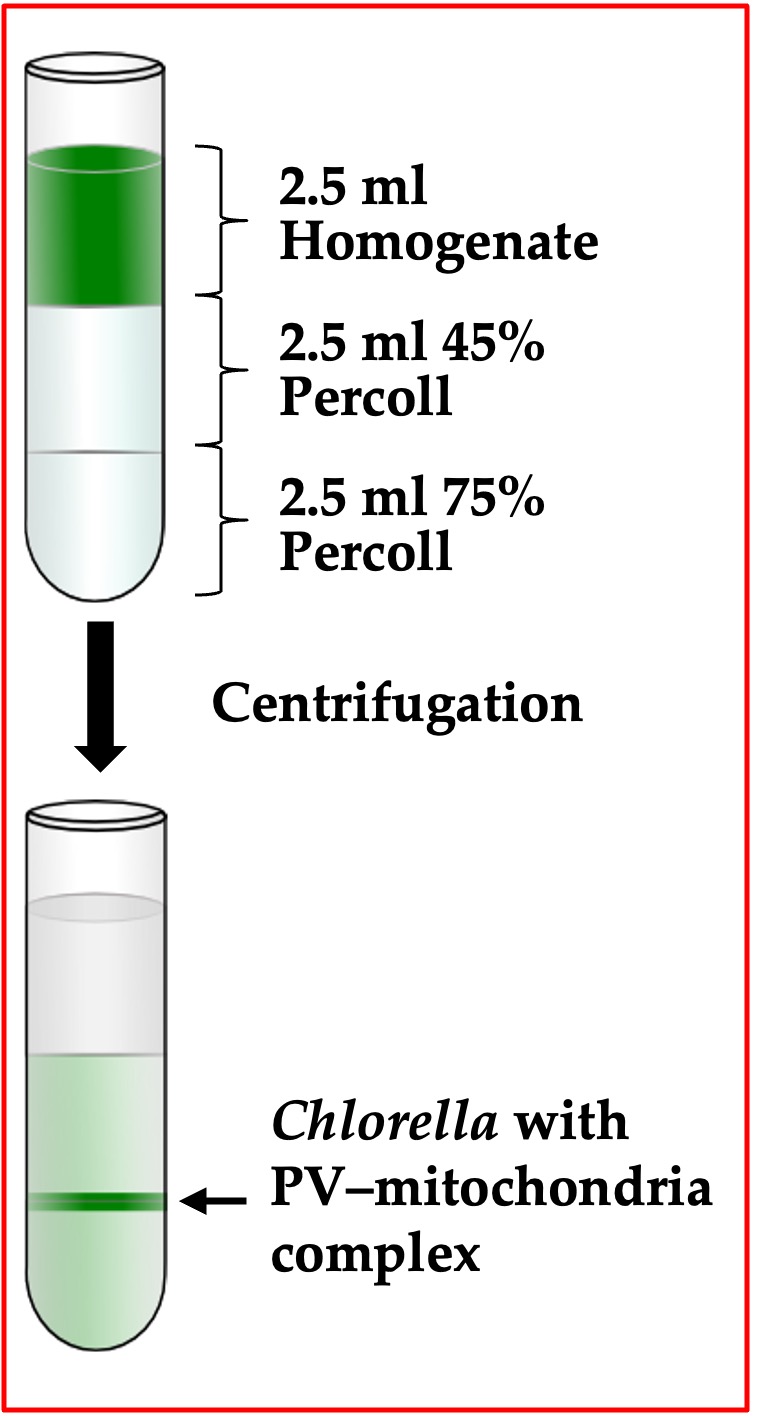

Supplement: Supplementary file 1 [file biomolecules-16-00561-s001.zip › Raw_images Fig1-5,7,8/Fig3_raw.jpg]

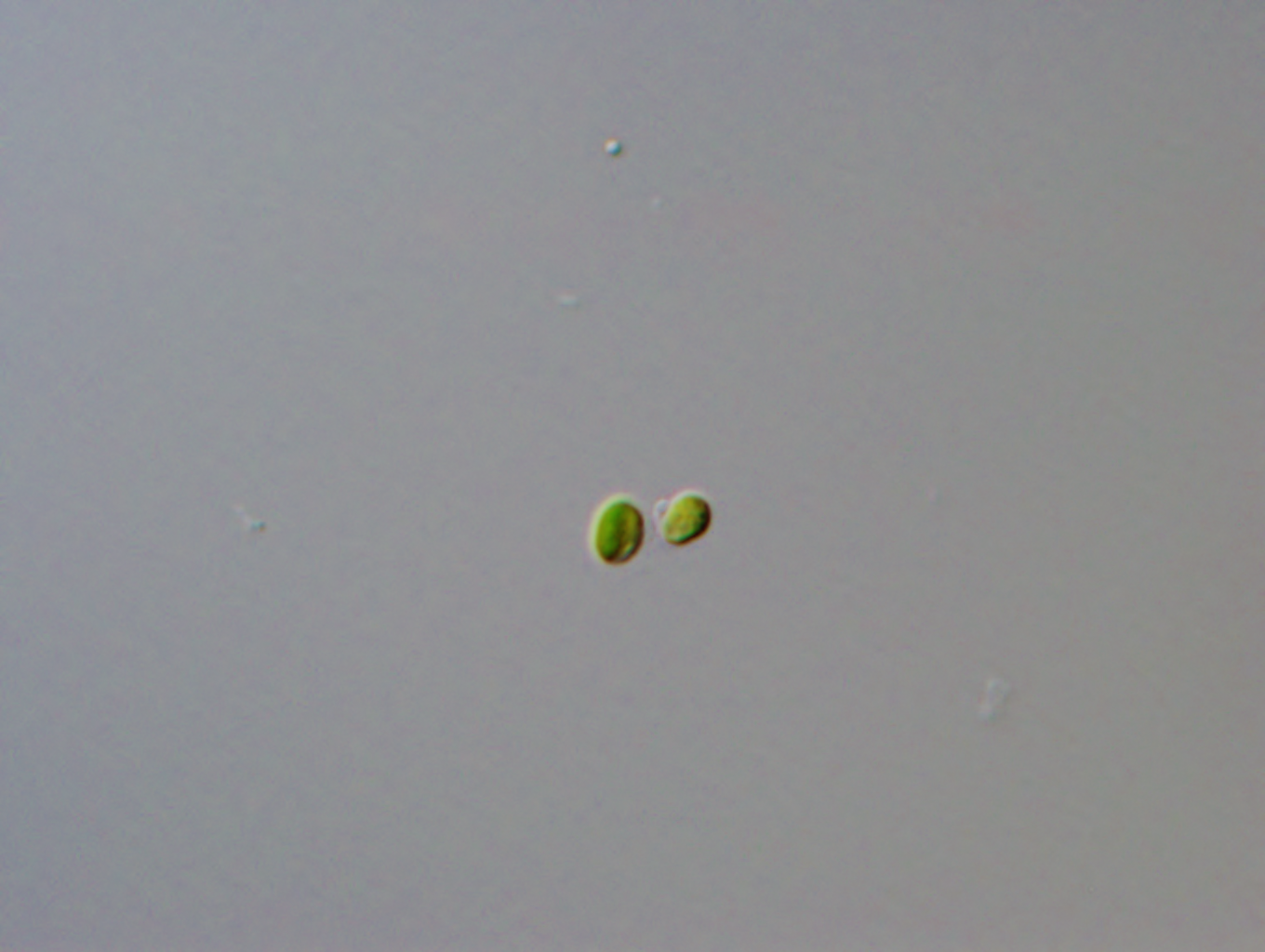

Supplement: Supplementary file 1 [file biomolecules-16-00561-s001.zip › Raw_images Fig1-5,7,8/Fig4A_raw.tif]

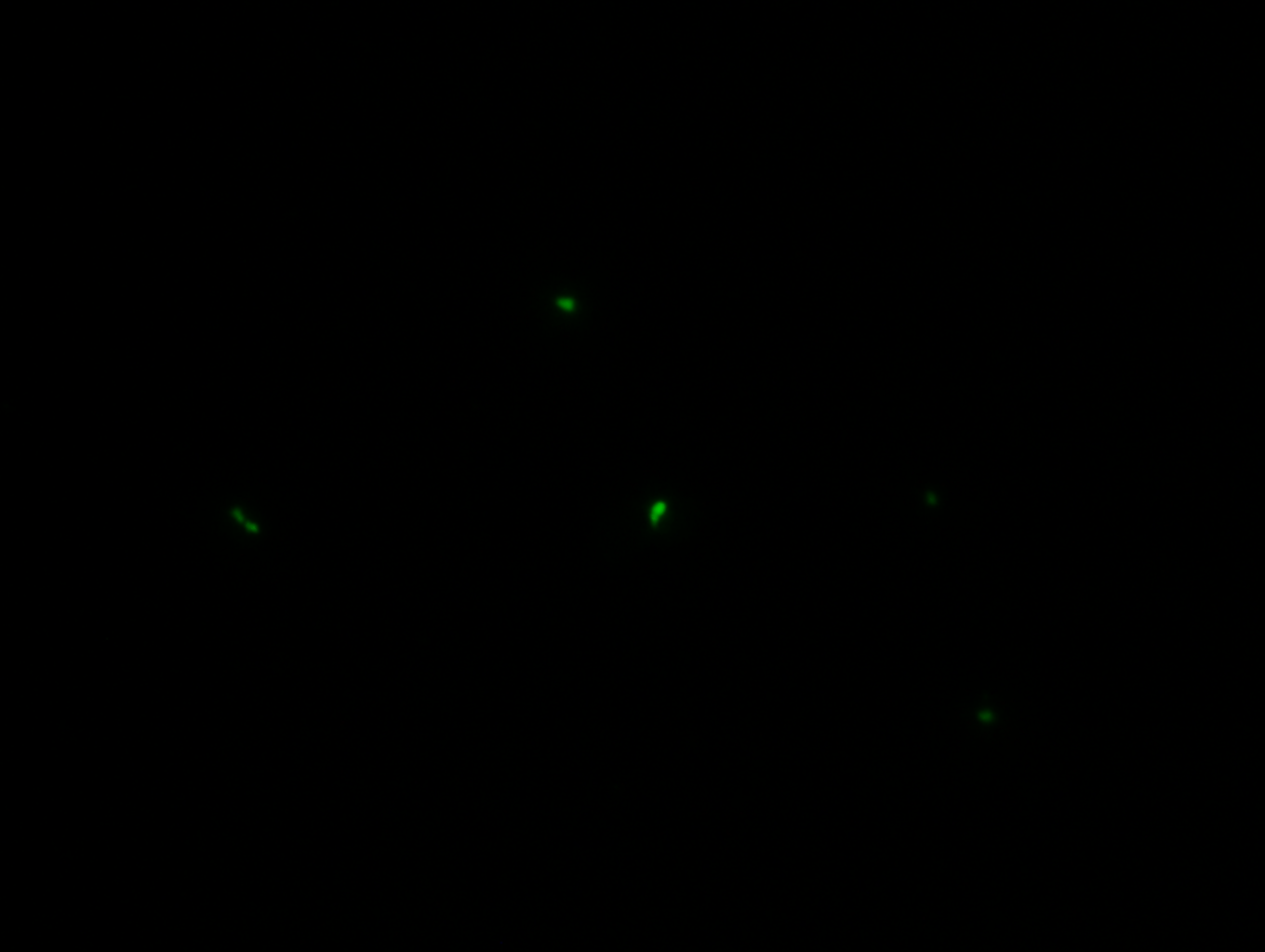

Supplement: Supplementary file 1 [file biomolecules-16-00561-s001.zip › Raw_images Fig1-5,7,8/Fig4B_raw.tif]

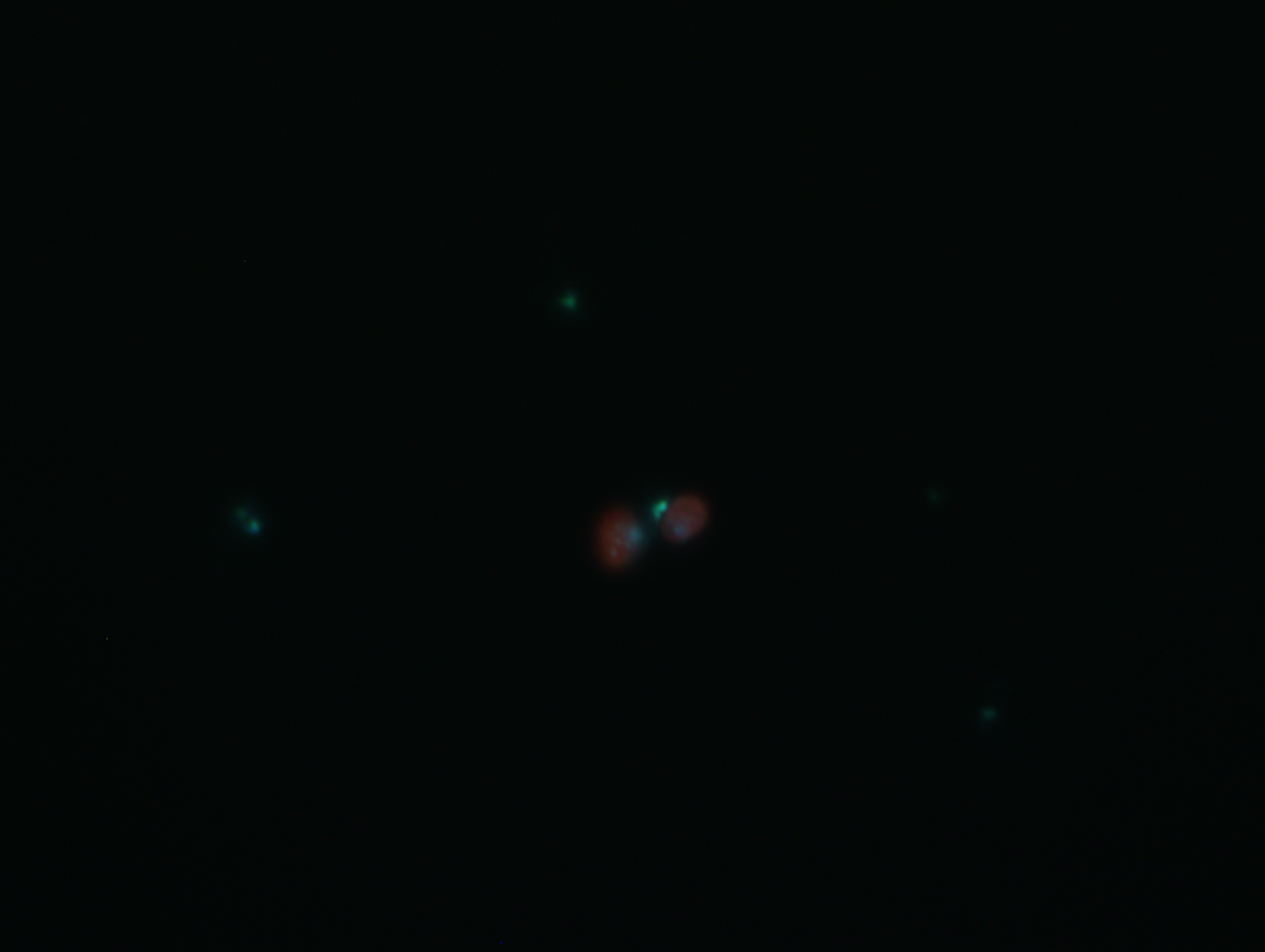

Supplement: Supplementary file 1 [file biomolecules-16-00561-s001.zip › Raw_images Fig1-5,7,8/Fig4C_raw.tif]

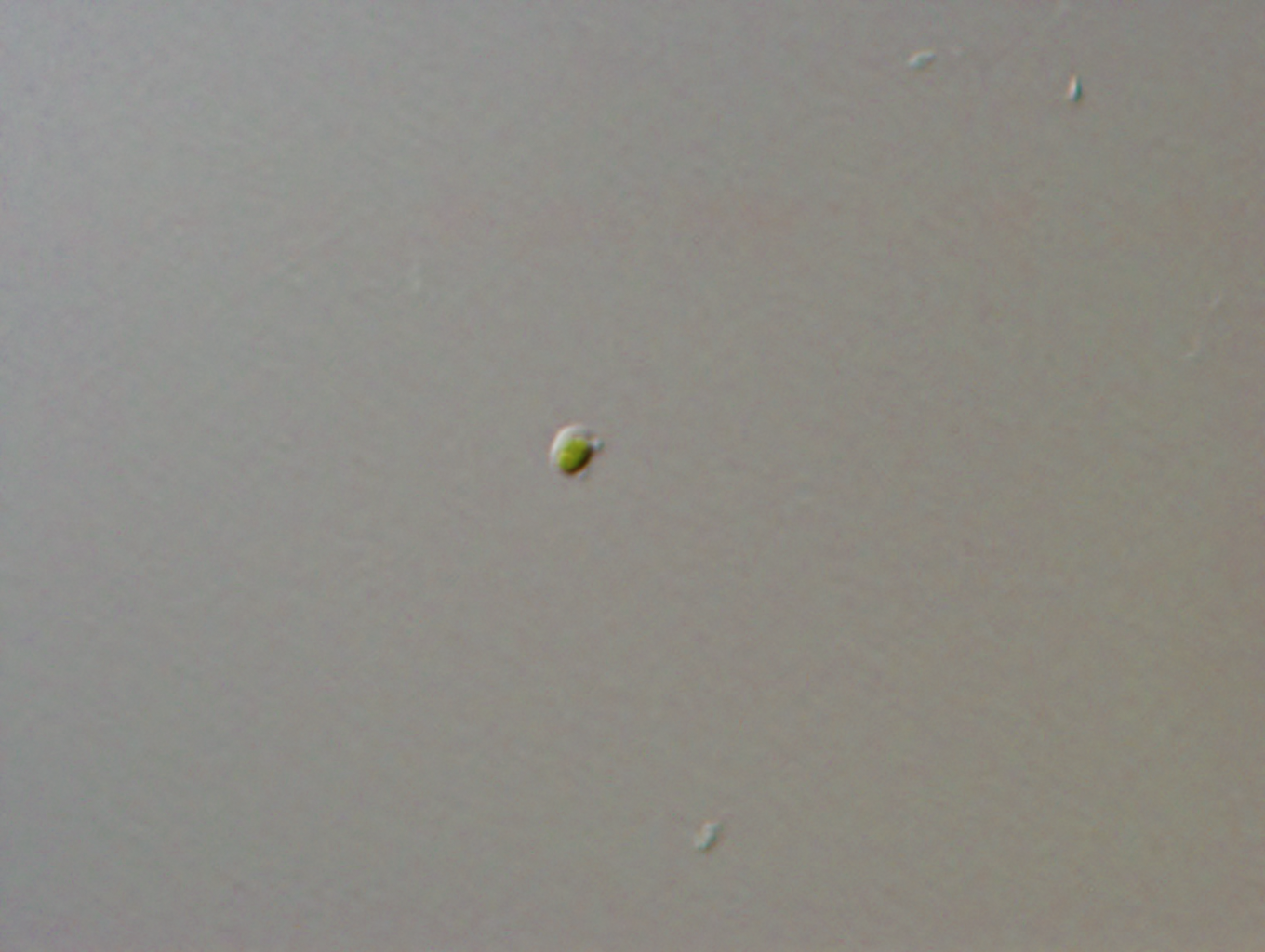

Supplement: Supplementary file 1 [file biomolecules-16-00561-s001.zip › Raw_images Fig1-5,7,8/Fig4E_raw.tif]

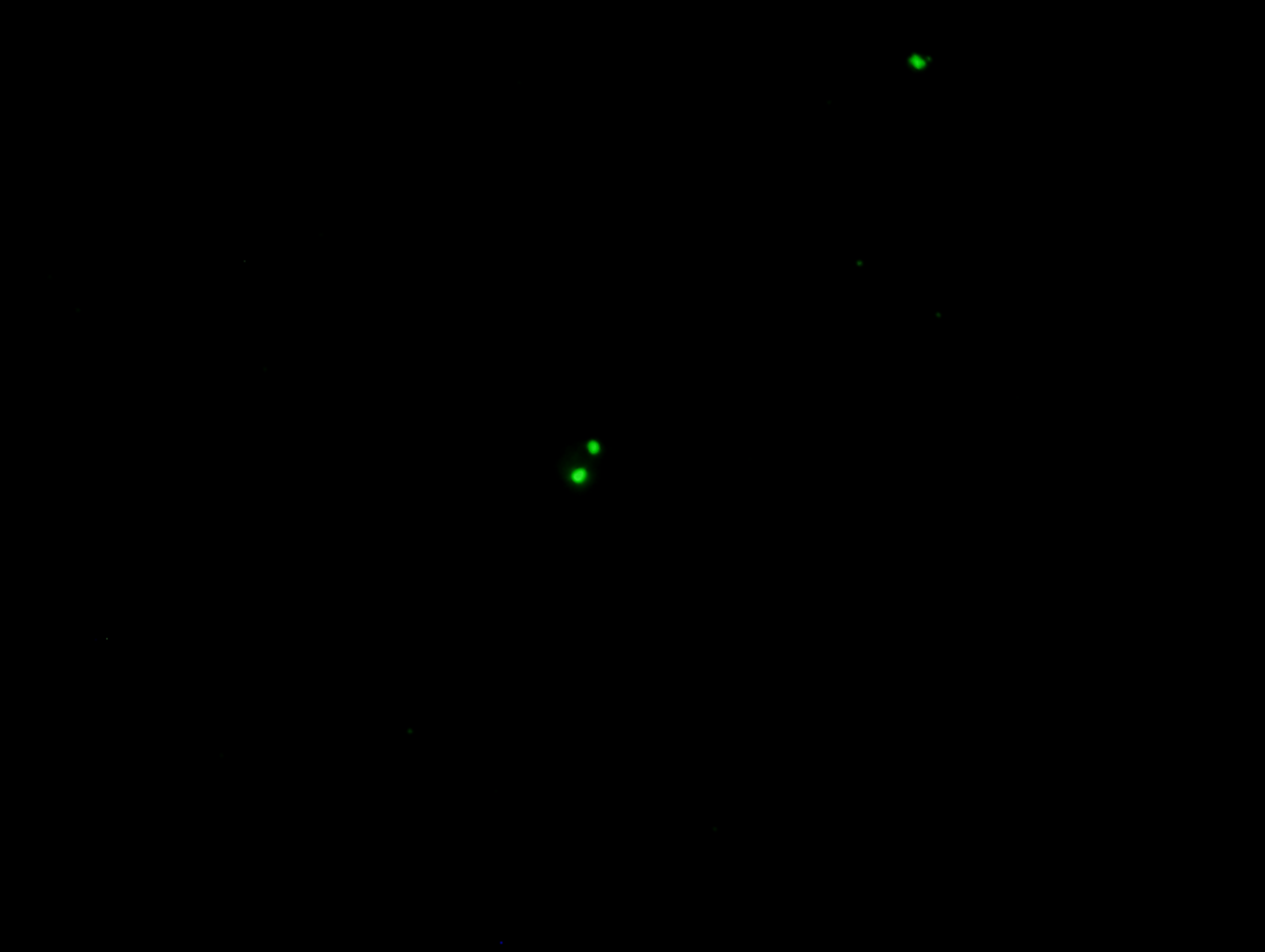

Supplement: Supplementary file 1 [file biomolecules-16-00561-s001.zip › Raw_images Fig1-5,7,8/Fig4F_raw.tif]

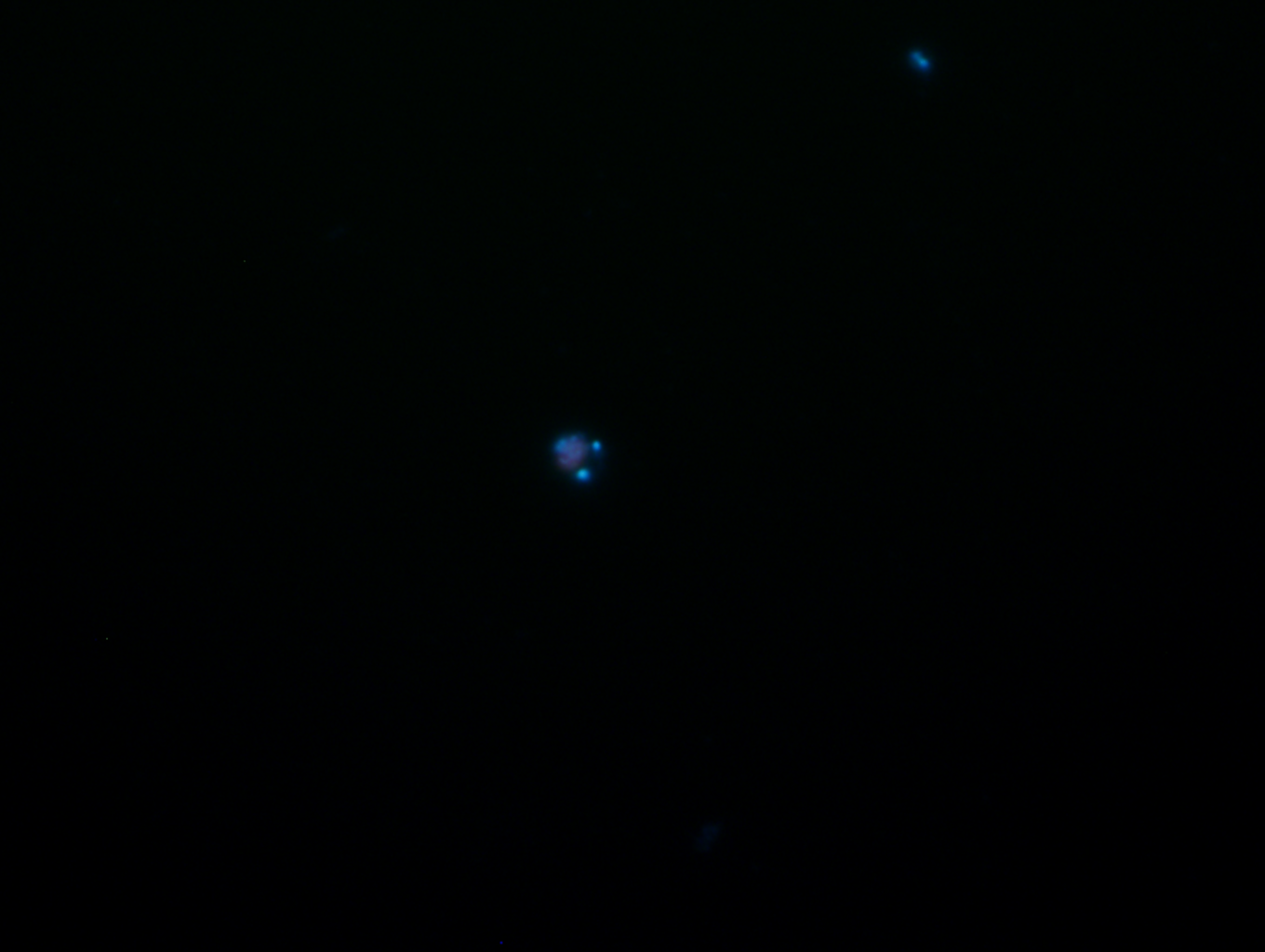

Supplement: Supplementary file 1 [file biomolecules-16-00561-s001.zip › Raw_images Fig1-5,7,8/Fig4G_raw.tif]

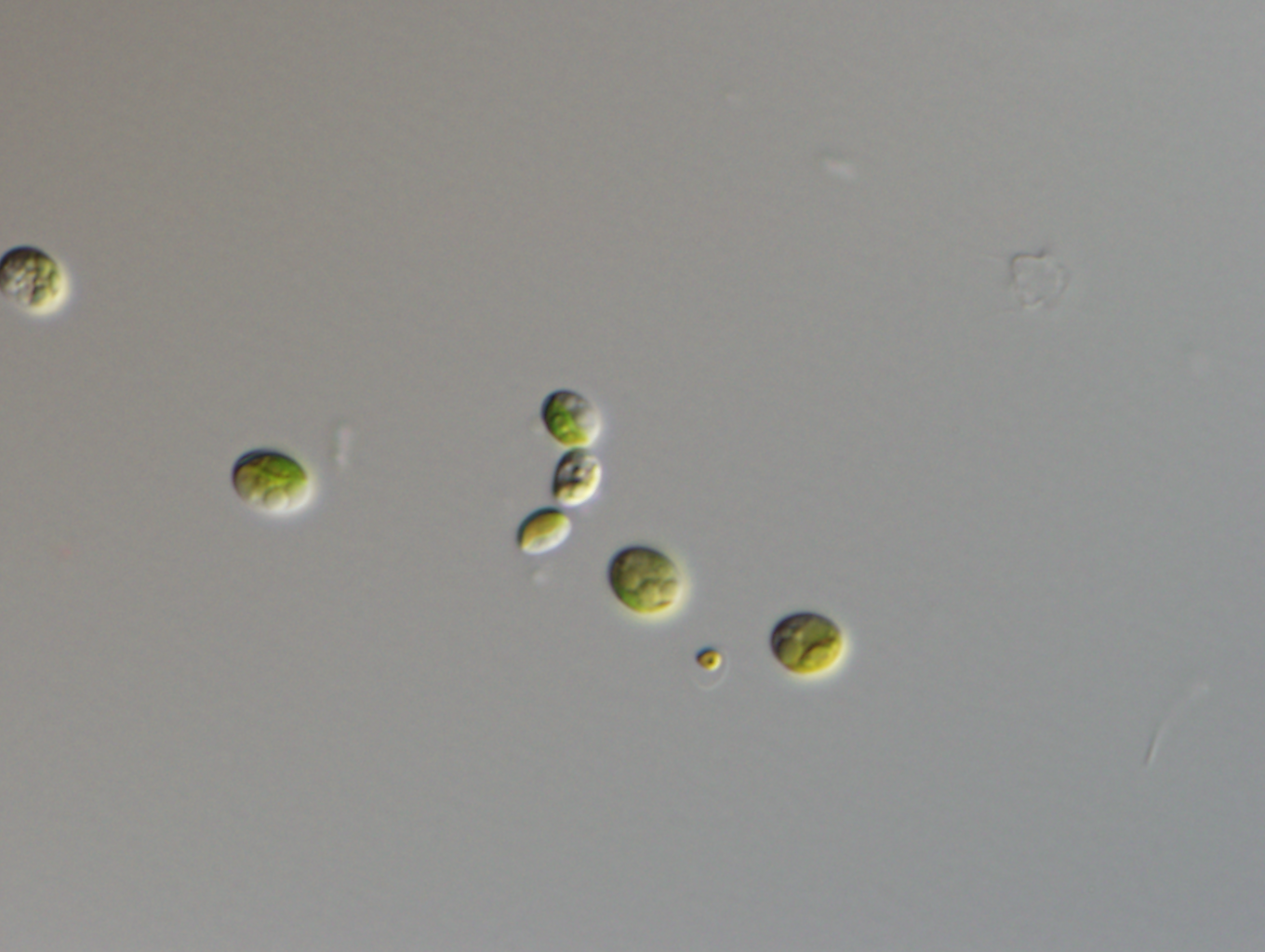

Supplement: Supplementary file 1 [file biomolecules-16-00561-s001.zip › Raw_images Fig1-5,7,8/Fig5A_raw.tif]

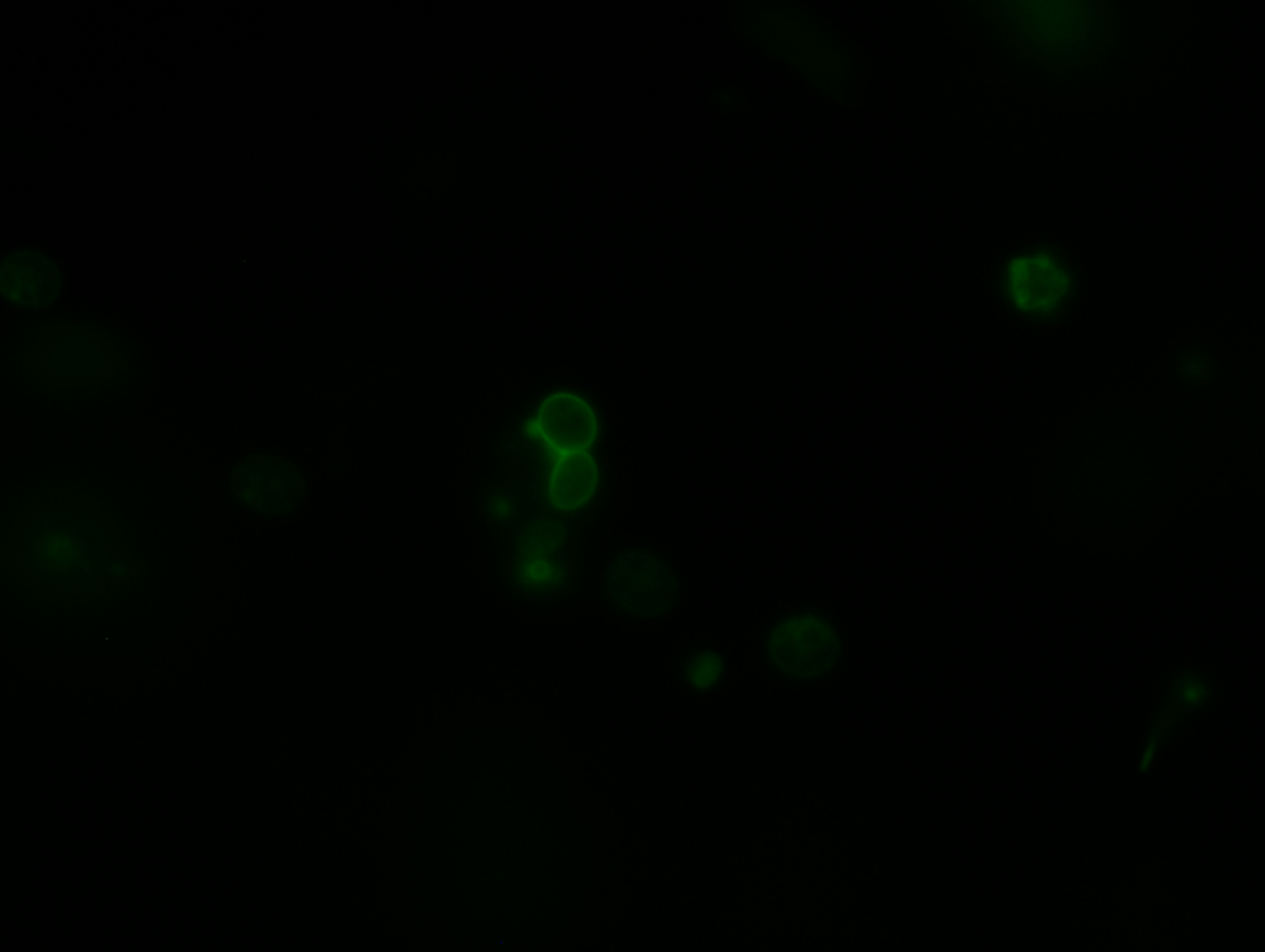

Supplement: Supplementary file 1 [file biomolecules-16-00561-s001.zip › Raw_images Fig1-5,7,8/Fig5B_raw.tif]

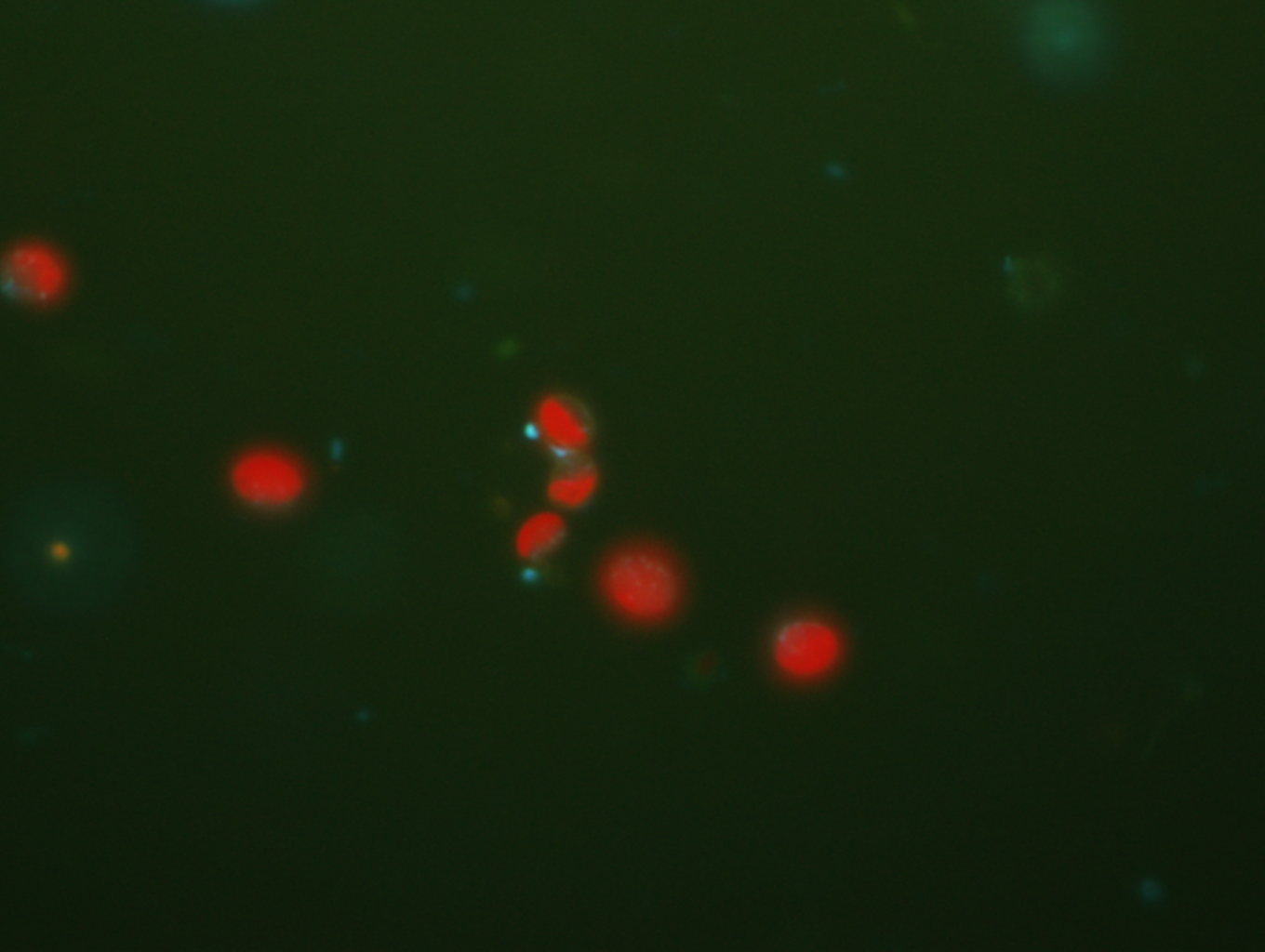

Supplement: Supplementary file 1 [file biomolecules-16-00561-s001.zip › Raw_images Fig1-5,7,8/Fig5C_raw.tif]

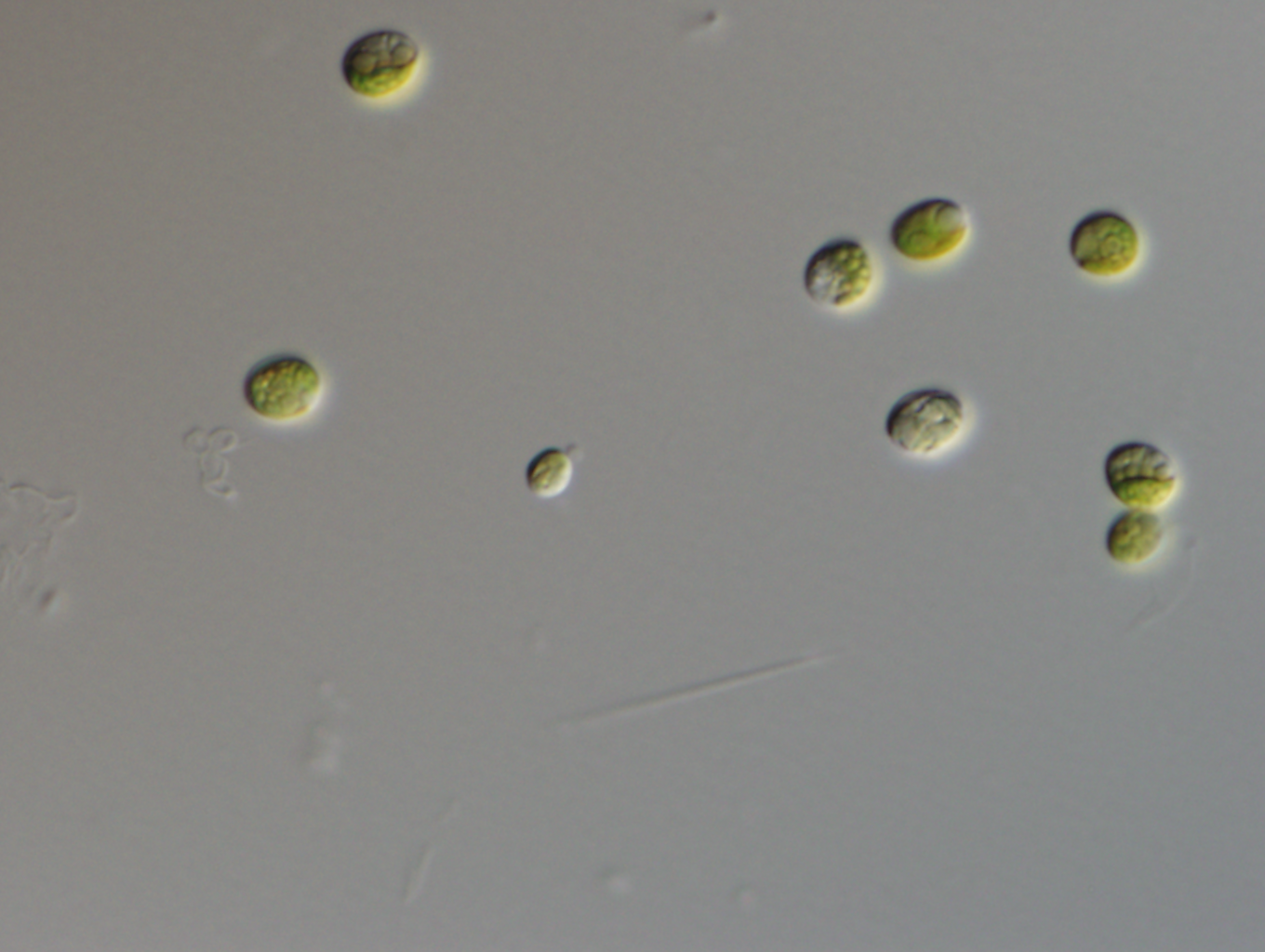

Supplement: Supplementary file 1 [file biomolecules-16-00561-s001.zip › Raw_images Fig1-5,7,8/Fig5E_raw.tif]

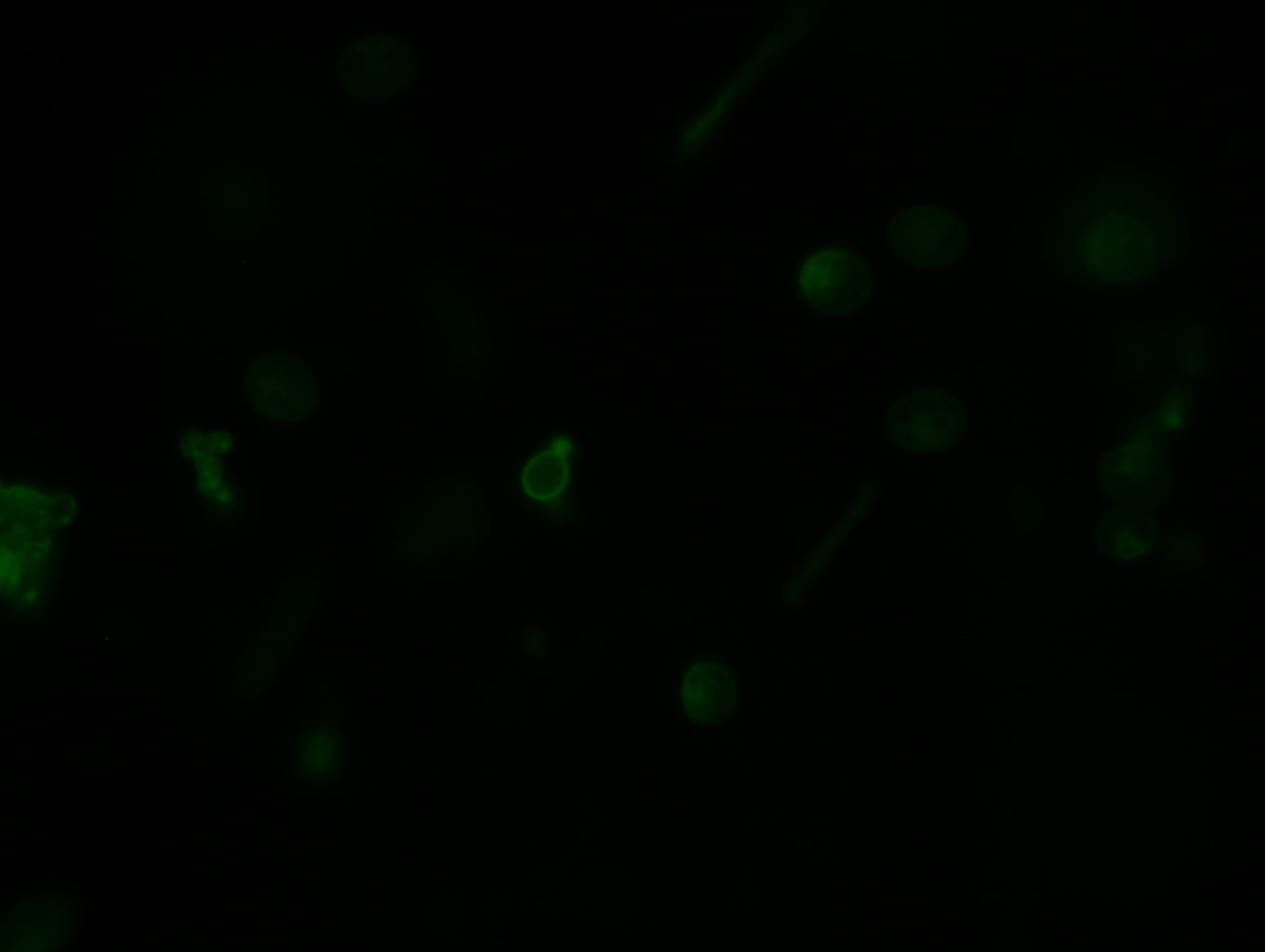

Supplement: Supplementary file 1 [file biomolecules-16-00561-s001.zip › Raw_images Fig1-5,7,8/Fig5F_raw.tif]

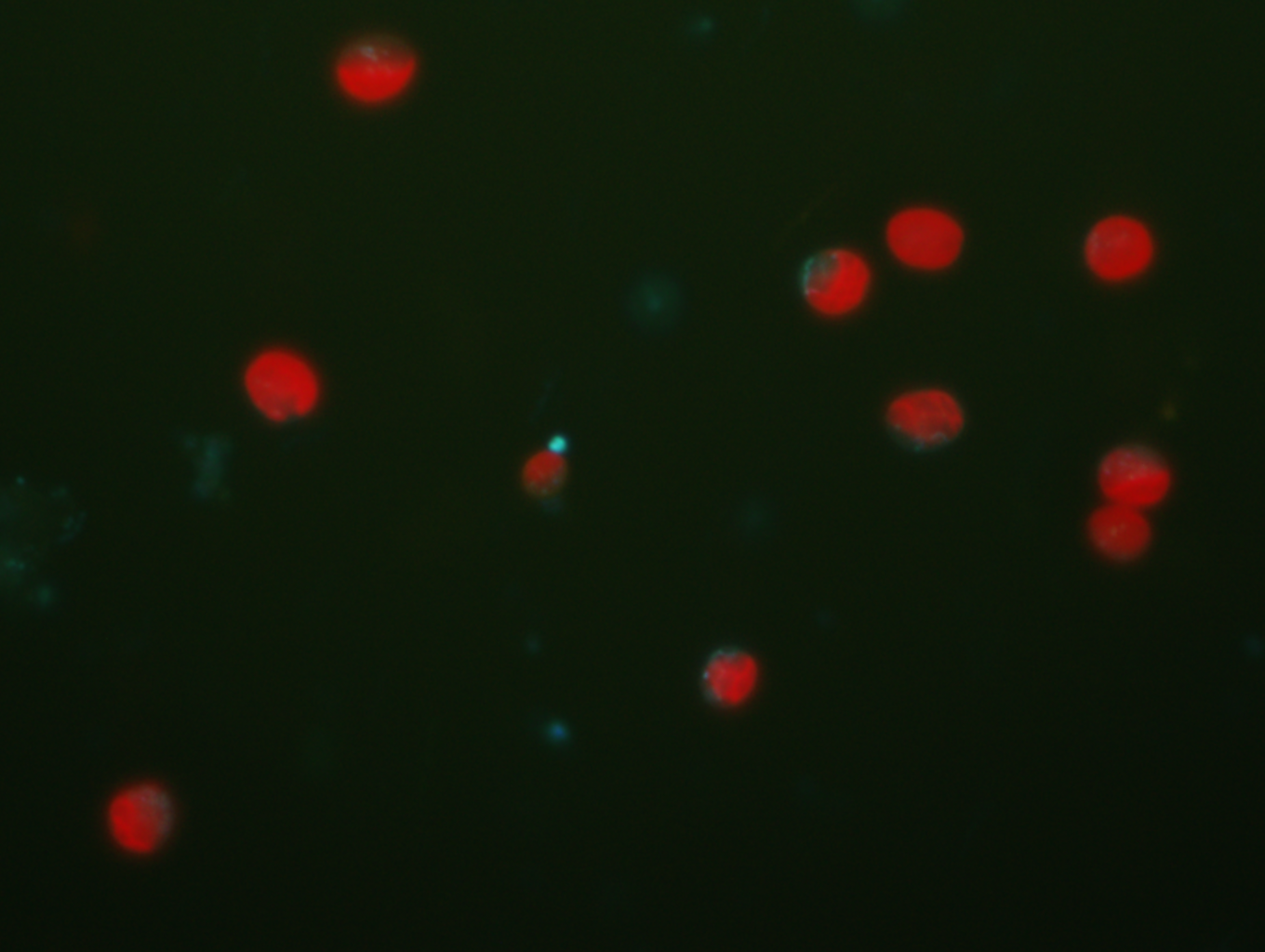

Supplement: Supplementary file 1 [file biomolecules-16-00561-s001.zip › Raw_images Fig1-5,7,8/Fig5G_raw.tif]

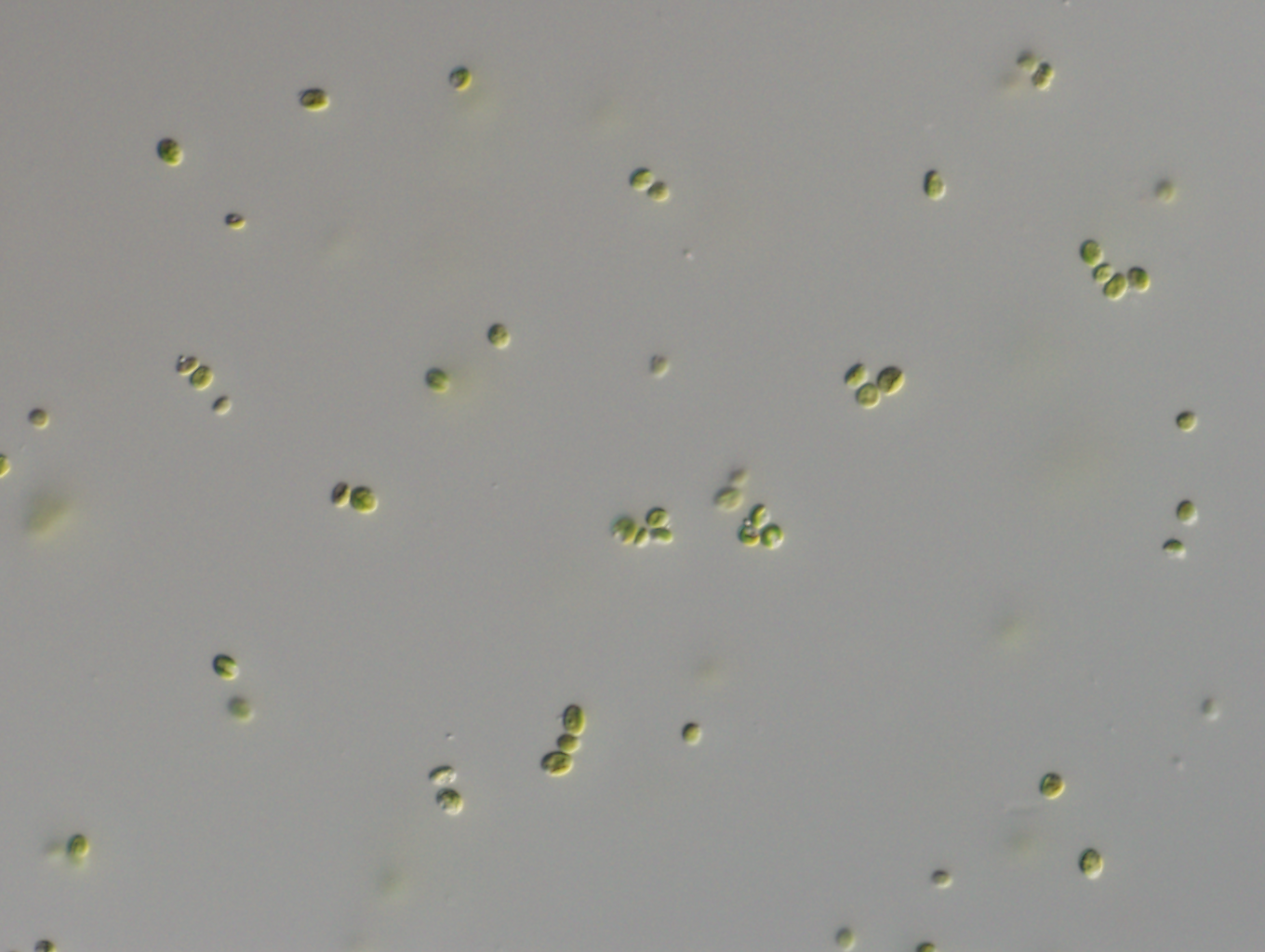

Supplement: Supplementary file 1 [file biomolecules-16-00561-s001.zip › Raw_images Fig1-5,7,8/Fig7A_raw.tif]

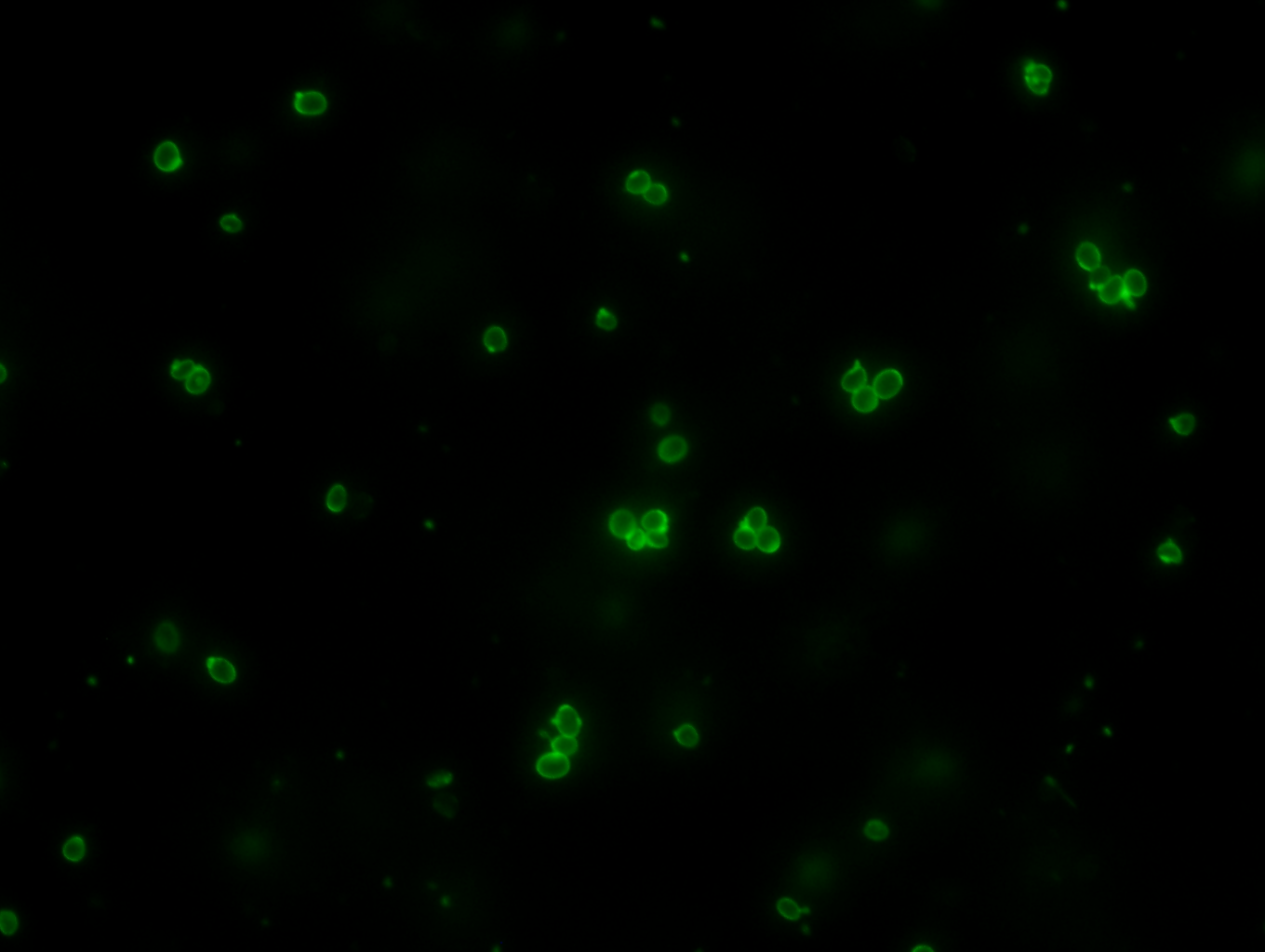

Supplement: Supplementary file 1 [file biomolecules-16-00561-s001.zip › Raw_images Fig1-5,7,8/Fig7B_raw.tif]

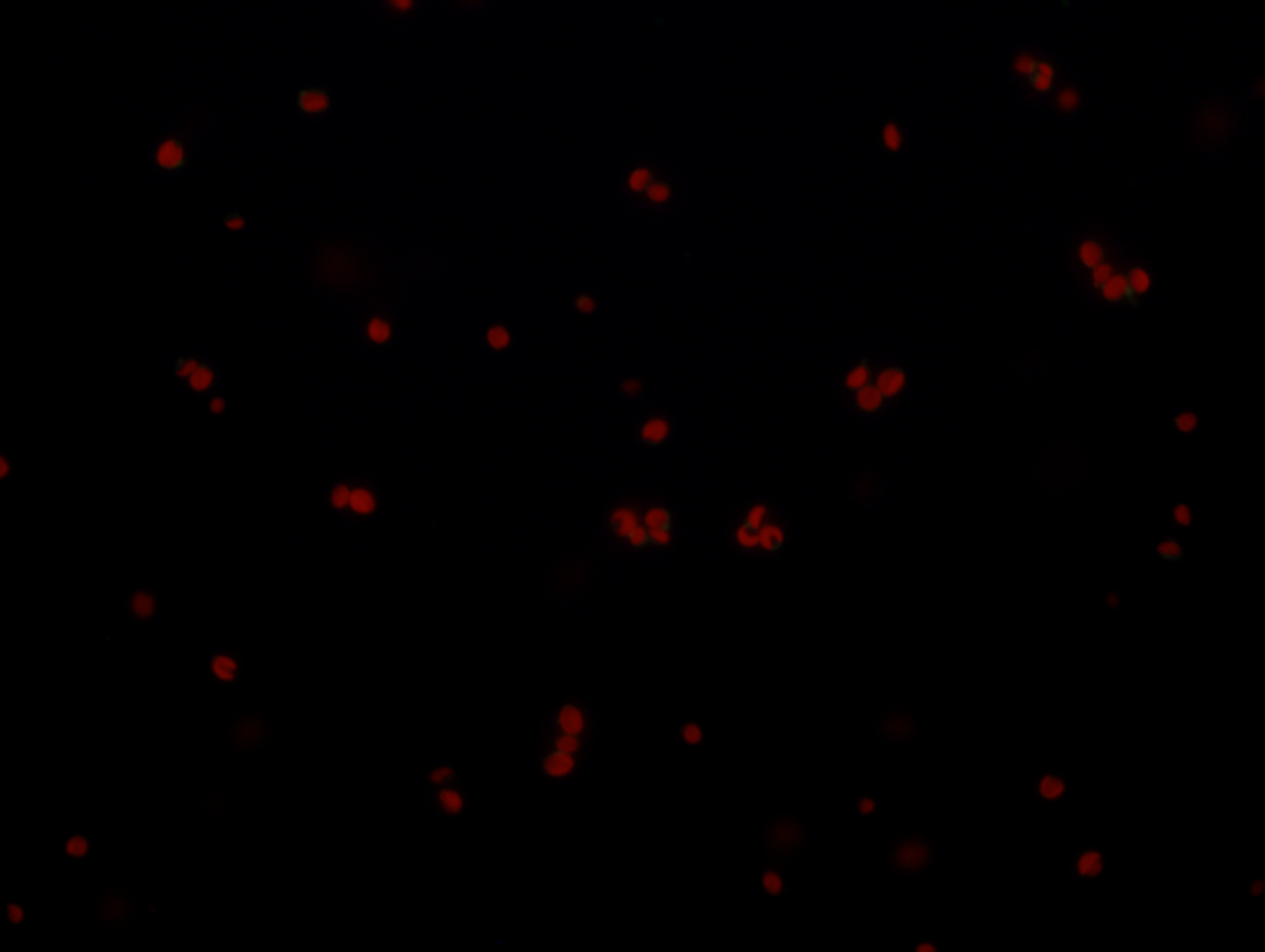

Supplement: Supplementary file 1 [file biomolecules-16-00561-s001.zip › Raw_images Fig1-5,7,8/Fig7C_raw.tif]

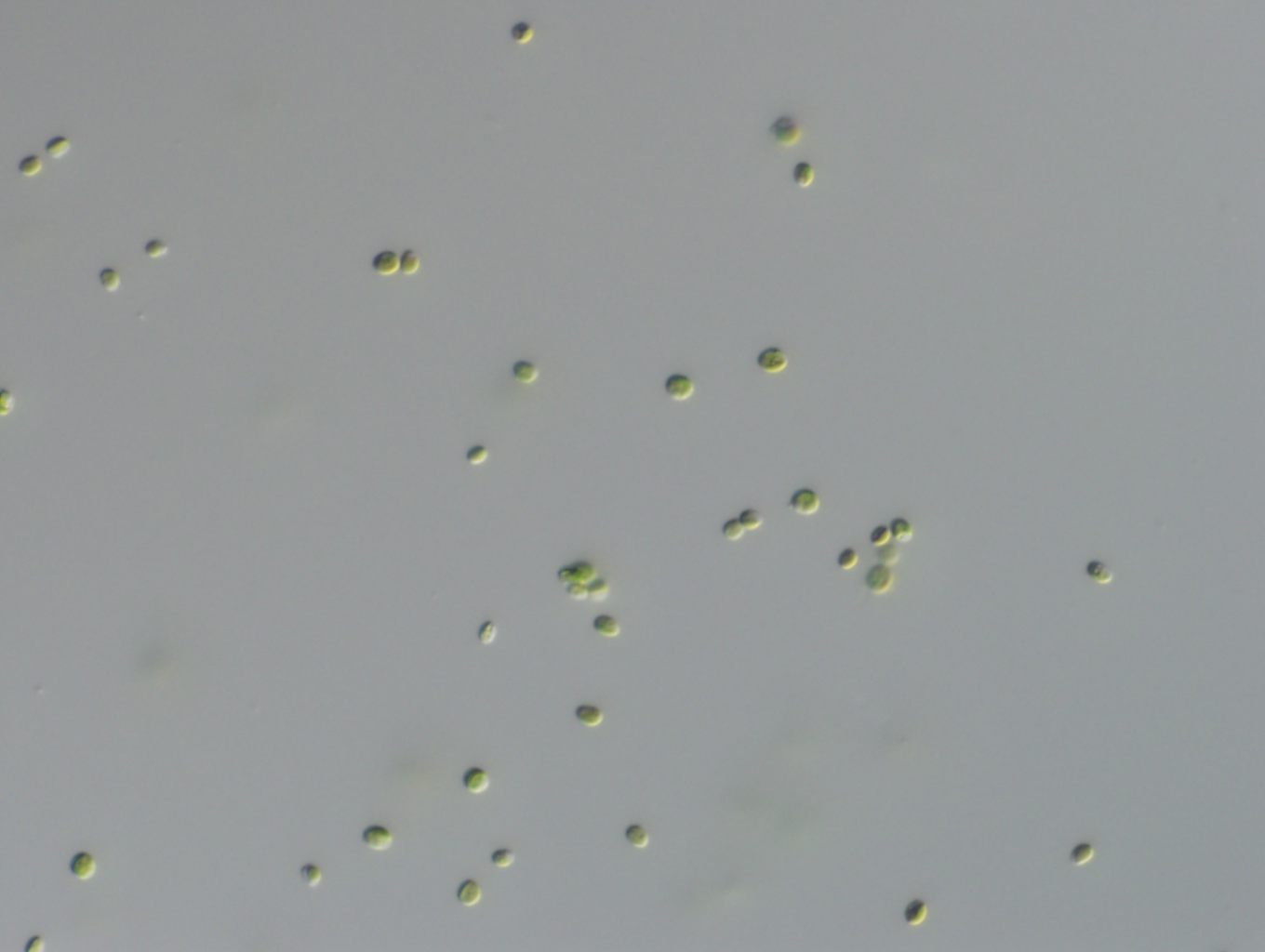

Supplement: Supplementary file 1 [file biomolecules-16-00561-s001.zip › Raw_images Fig1-5,7,8/Fig7D_raw.tif]

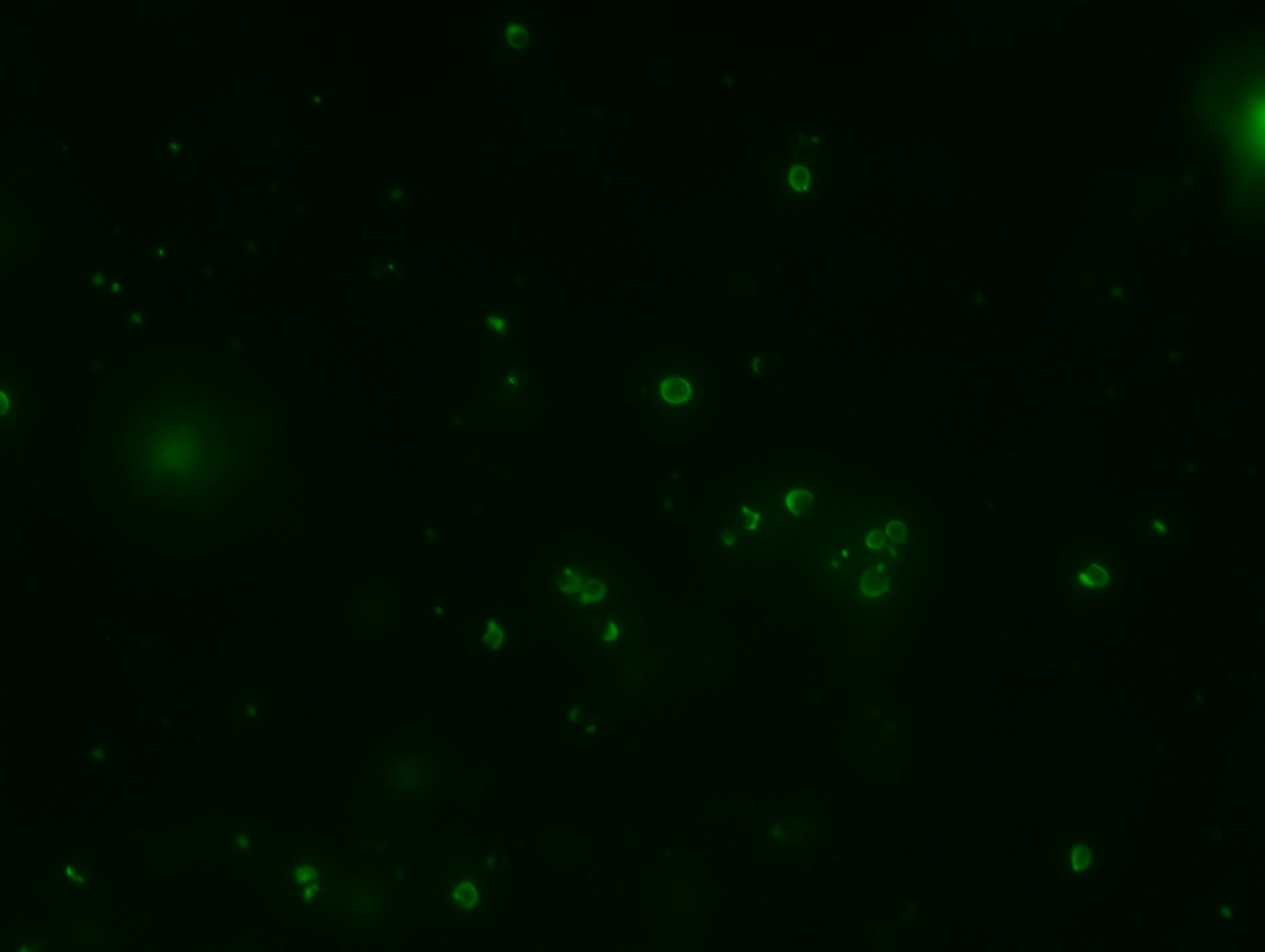

Supplement: Supplementary file 1 [file biomolecules-16-00561-s001.zip › Raw_images Fig1-5,7,8/Fig7E_raw.tif]

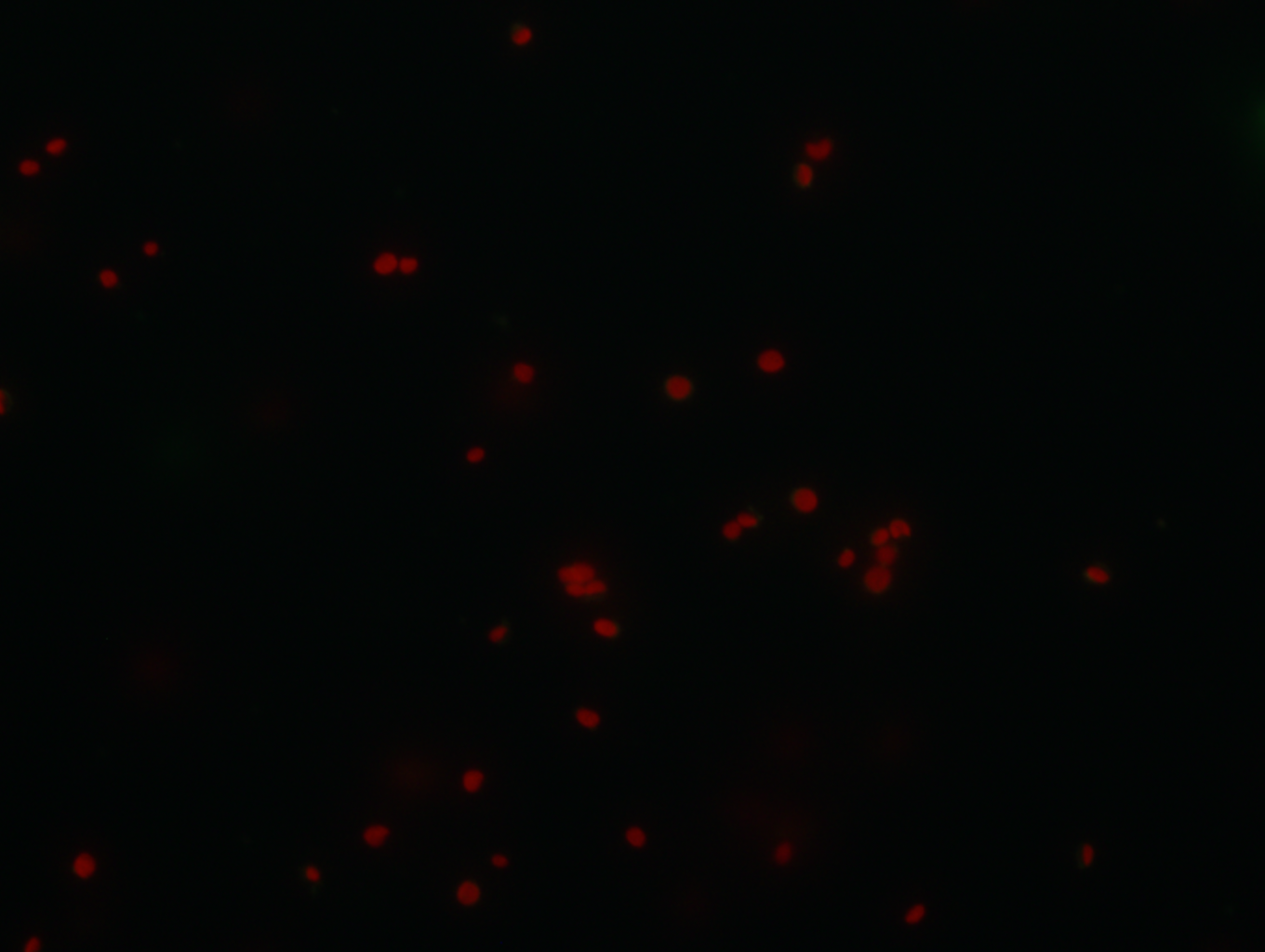

Supplement: Supplementary file 1 [file biomolecules-16-00561-s001.zip › Raw_images Fig1-5,7,8/Fig7F_raw.tif]

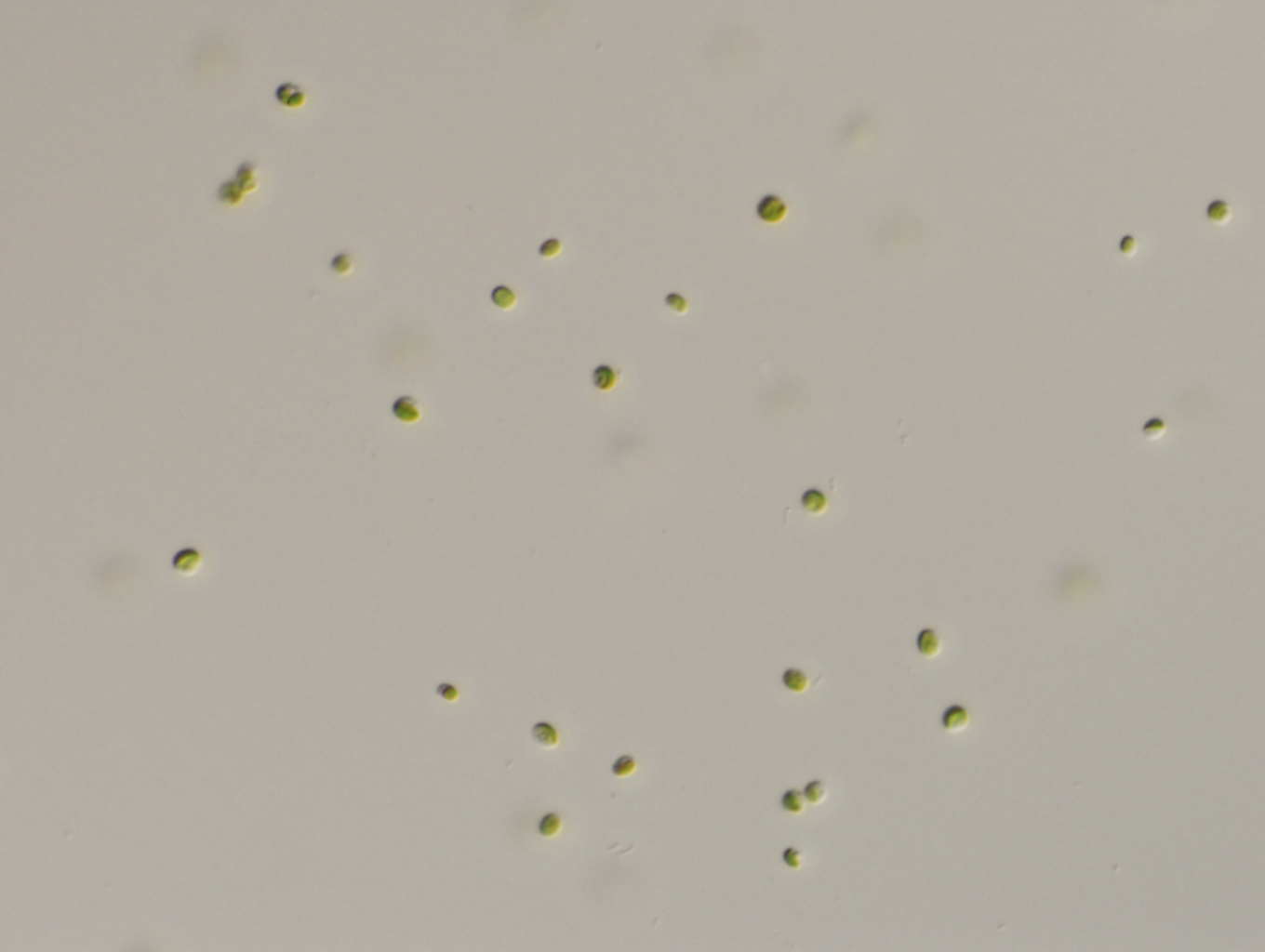

Supplement: Supplementary file 1 [file biomolecules-16-00561-s001.zip › Raw_images Fig1-5,7,8/Fig7G_raw.tif]

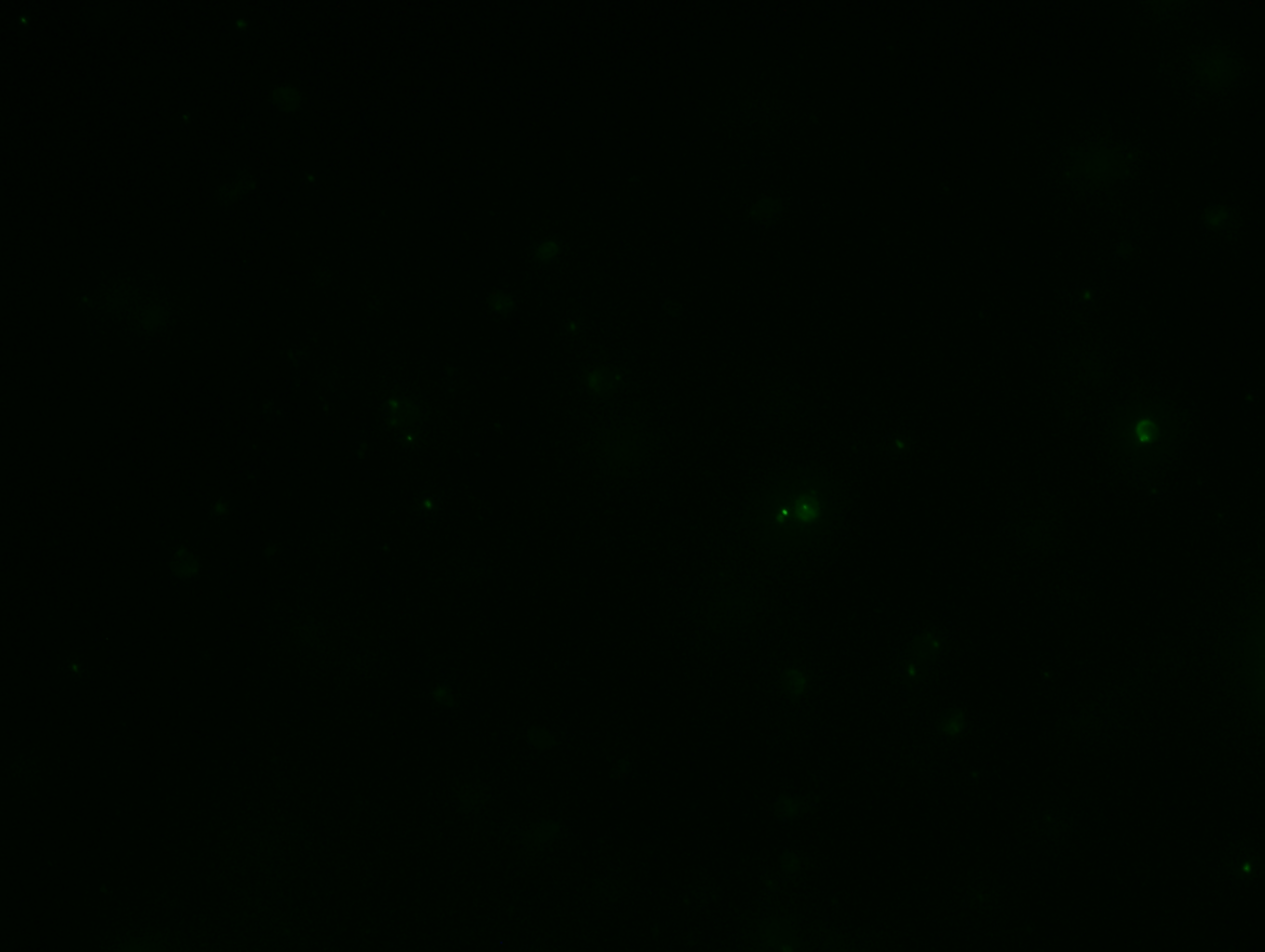

Supplement: Supplementary file 1 [file biomolecules-16-00561-s001.zip › Raw_images Fig1-5,7,8/Fig7H_raw.tif]

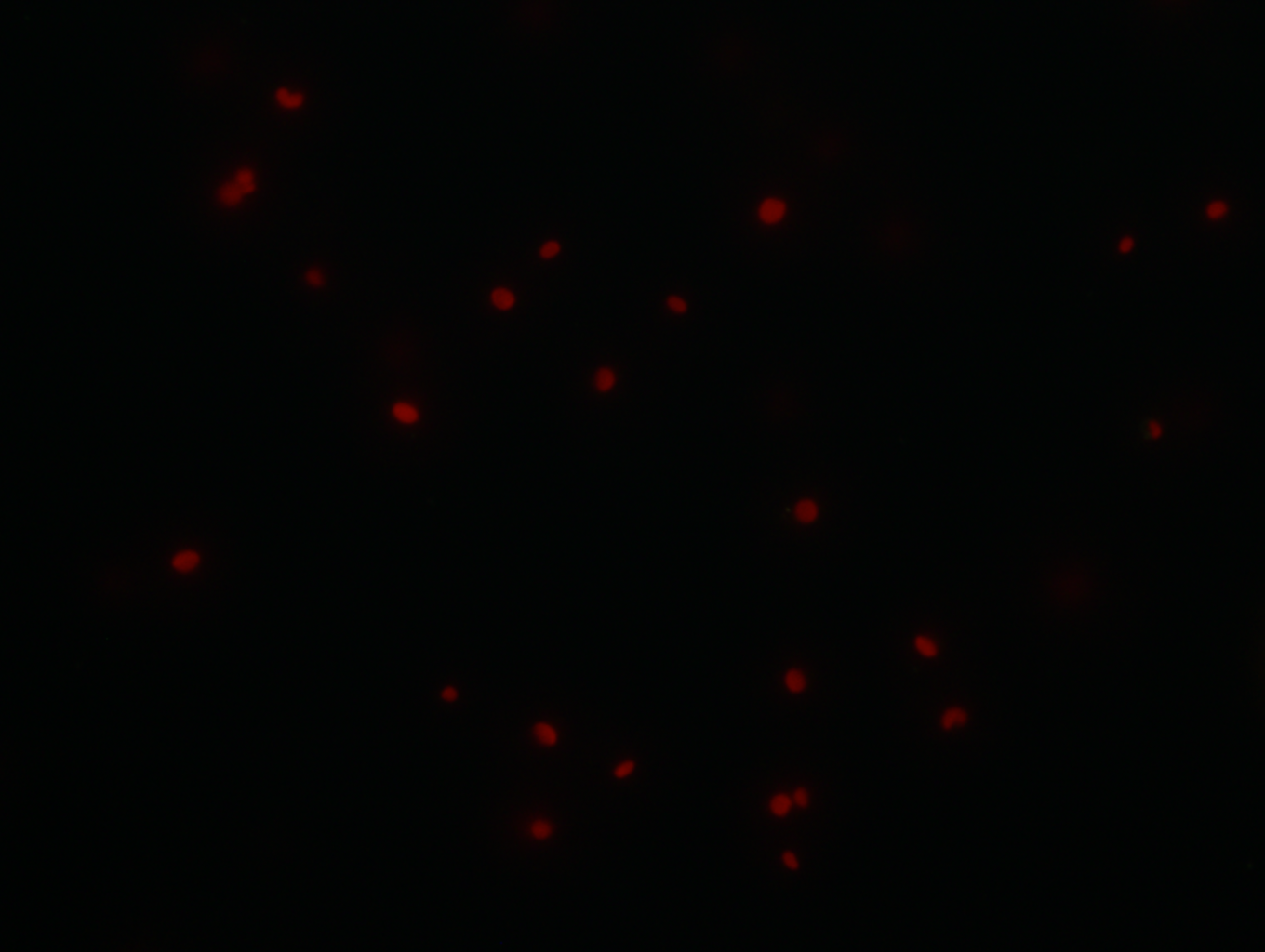

Supplement: Supplementary file 1 [file biomolecules-16-00561-s001.zip › Raw_images Fig1-5,7,8/Fig7I_raw.tif]

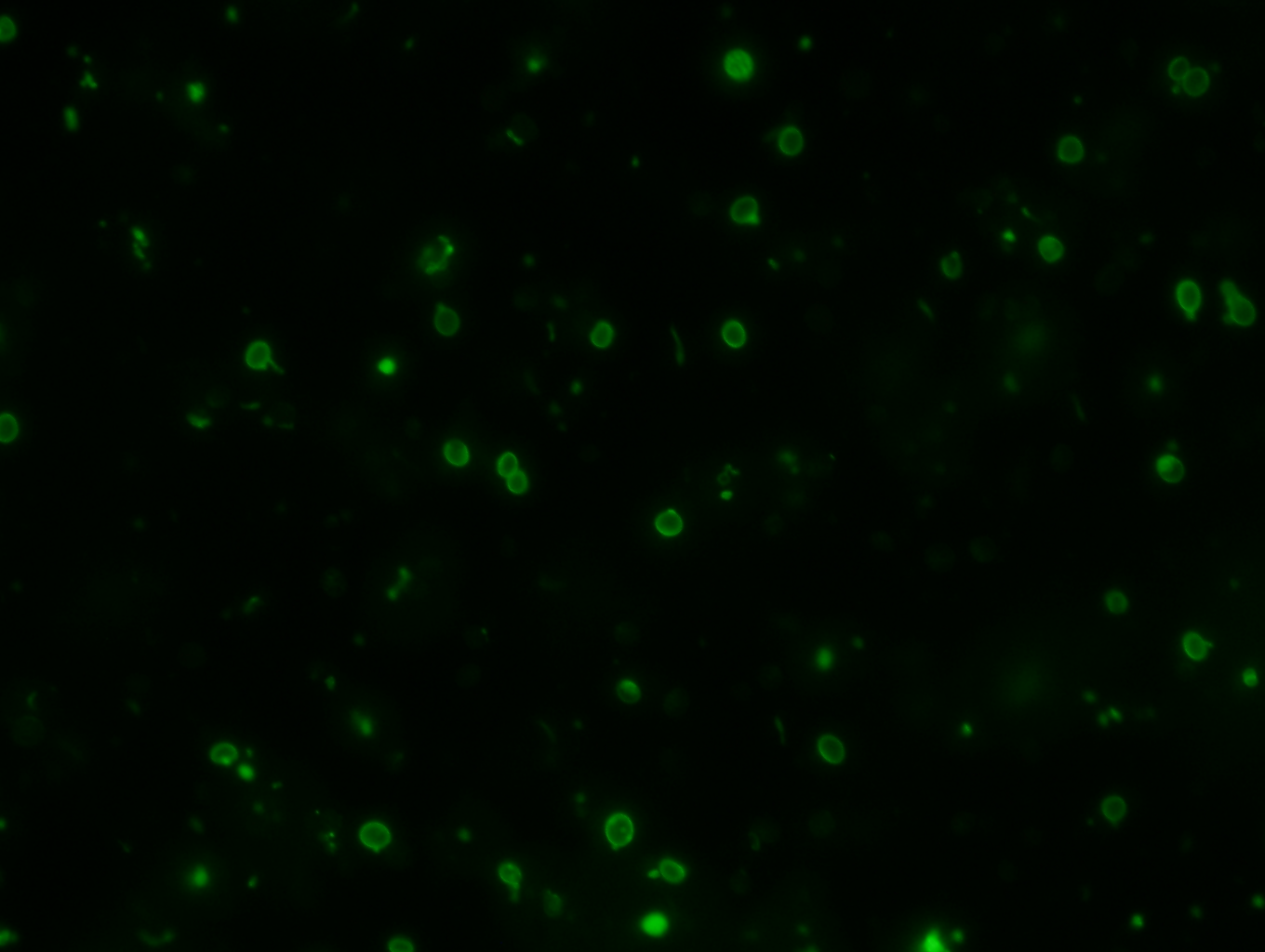

Supplement: Supplementary file 1 [file biomolecules-16-00561-s001.zip › Raw_images Fig1-5,7,8/Fig8_Day0_BC5C_raw.tif]

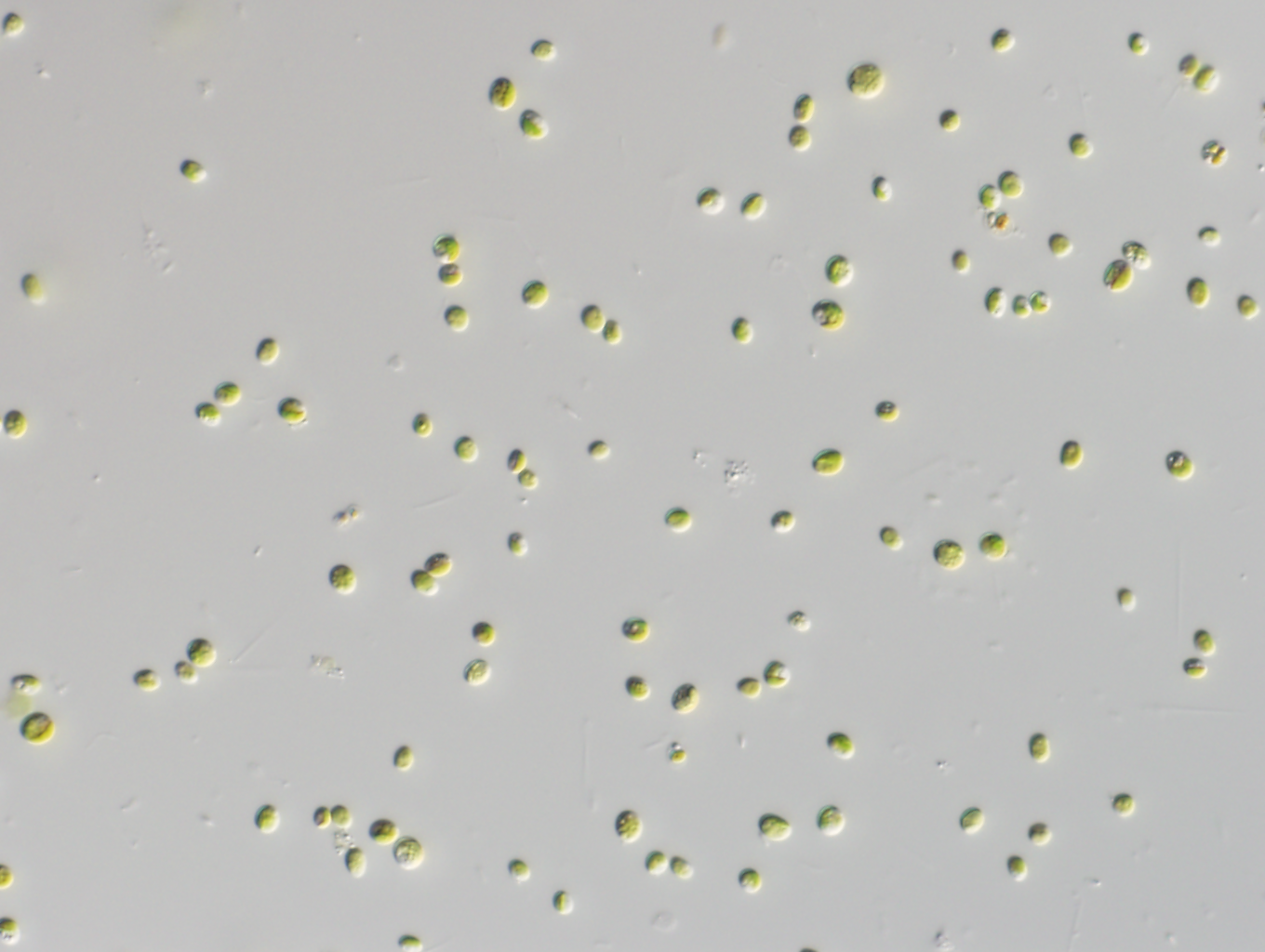

Supplement: Supplementary file 1 [file biomolecules-16-00561-s001.zip › Raw_images Fig1-5,7,8/Fig8_Day0_DIC_raw.tif]

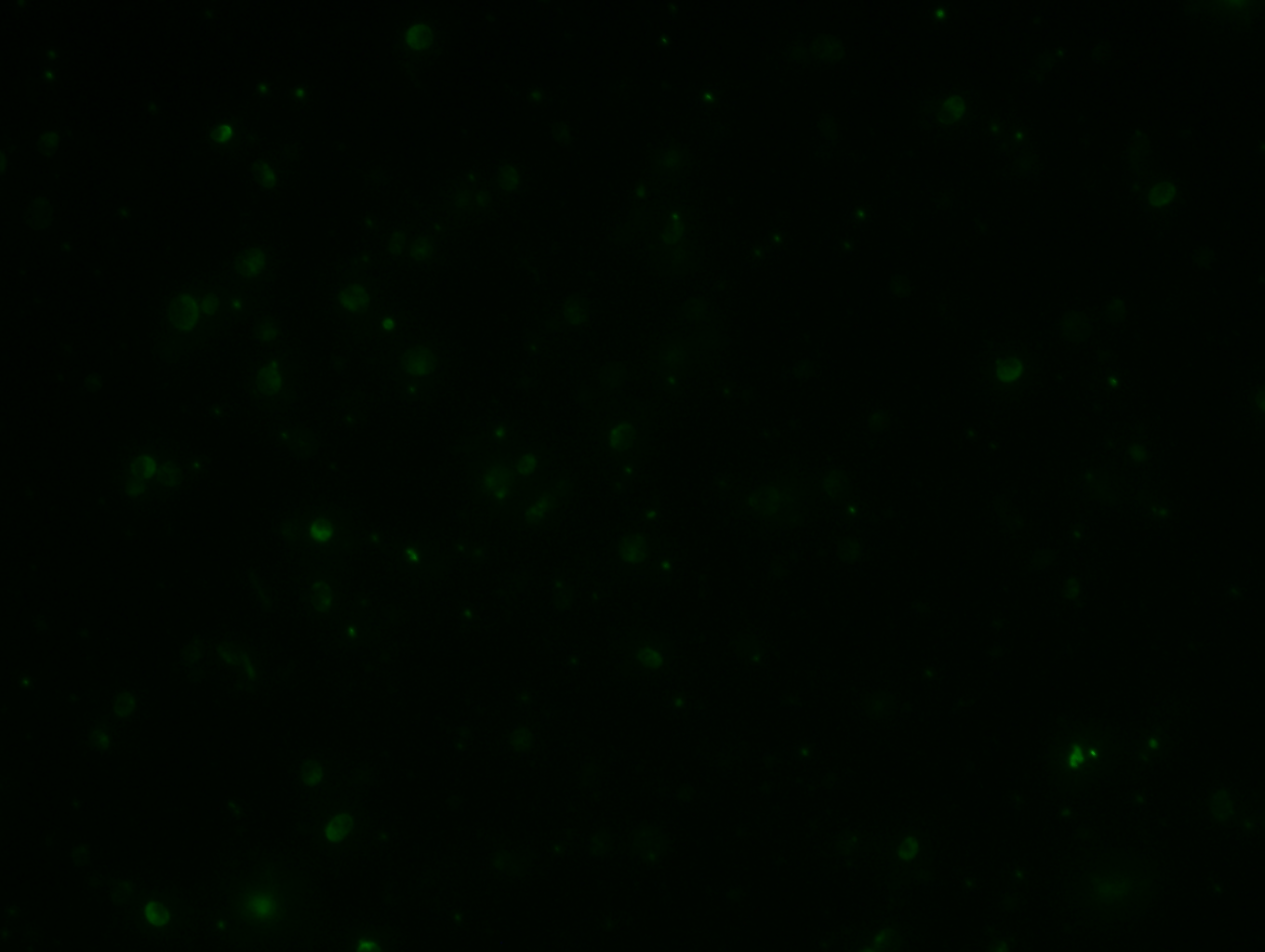

Supplement: Supplementary file 1 [file biomolecules-16-00561-s001.zip › Raw_images Fig1-5,7,8/Fig8_Day7_BC5C_raw.tif]

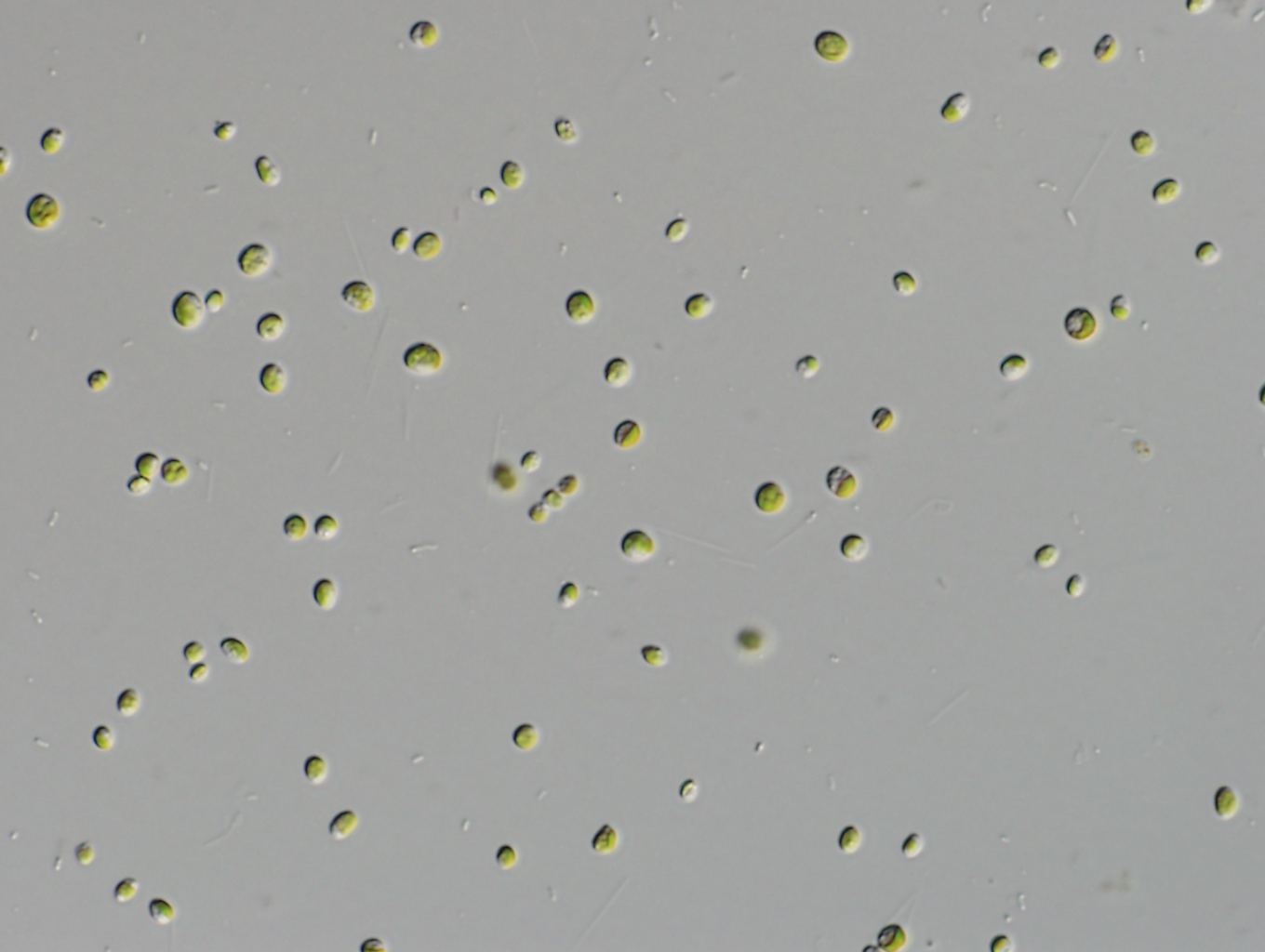

Supplement: Supplementary file 1 [file biomolecules-16-00561-s001.zip › Raw_images Fig1-5,7,8/Fig8_Day7_DIC_raw.tif]
